# Chloroplast magnesium transporters play essential but differential roles in maintaining magnesium homeostasis

**DOI:** 10.3389/fpls.2023.1221436

**Published:** 2023-08-23

**Authors:** Emilija Dukic, Kim A. van Maldegem, Kashif Mohd Shaikh, Kento Fukuda, Mats Töpel, Katalin Solymosi, Jonna Hellsten, Thomas Hesselhøj Hansen, Søren Husted, John Higgins, Satoshi Sano, Sumio Ishijima, Cornelia Spetea

**Affiliations:** ^1^ Department of Biological and Environmental Sciences, University of Gothenburg, Gothenburg, Sweden; ^2^ Graduate School of Life and Environmental Sciences, Kyoto Prefectural University, Kyoto, Japan; ^3^ Department of Marine Sciences, University of Gothenburg, Gothenburg, Sweden; ^4^ IVL Swedish Environmental Research Institute, Gothenburg, Sweden; ^5^ Department of Plant Anatomy, ELTE Eötvös Loránd University, Budapest, Hungary; ^6^ Copenhagen Plant Science Centre, Department of Plant and Environmental Sciences, University of Copenhagen, Copenhagen, Denmark; ^7^ Department of Geosciences, Princeton University, Princeton, NJ, United States

**Keywords:** *Arabidopsis thaliana*, *Chlamydomonas reinhardtii*, chloroplast, magnesium homeostasis, magnesium transporter, chlorophyll fluorescence, photosynthesis

## Abstract

Magnesium (Mg^2+^) is essential for photosynthesis in the chloroplasts of land plants and algae. Being the central ion of chlorophyll, cofactor and activator of many photosynthetic enzymes including RuBisCO, magnesium-deficient plants may suffer from leaf chlorosis symptoms and retarded growth. Therefore, the chloroplast Mg^2+^ concentration is tightly controlled by magnesium transport proteins. Recently, three different transporters from two distinct families have been identified in the chloroplast inner envelope of the model plant *Arabidopsis thaliana*: MGT10, MGR8, and MGR9. Here, we assess the individual roles of these three proteins in maintaining chloroplast Mg^2+^ homeostasis and regulating photosynthesis, and if their role is conserved in the model green alga *Chlamydomonas reinhardtii*. Phylogenetic analysis and heterologous expression revealed that the CorC-like MGR8 and MGR9 transport Mg^2+^ by a different mechanism than the CorA-like MGT10. *MGR8* and *MGT10* genes are highest expressed in leaves, indicating a function in chloroplast Mg^2+^ transport. MGR9 is important for chloroplast function and plant adaptation in conditions of deficiency or excess of Mg^2+^. Transmission electron microscopy indicated that MGT10 plays a differential role in thylakoid stacking than MGR8 and MGR9. Furthermore, we report that MGR8, MGR9, and MGT10 are involved in building up the pH gradient across the thylakoid membrane and activating photoprotection in conditions of excess light, however the mechanism has not been resolved yet. While there are no chloroplast MGR-like transporters in Chlamydomonas, we show that MRS4 is a homolog of MGT10, that is required for photosynthesis and cell growth. Taken together, our findings reveal that the studied Mg^2+^ transporters play essential but differential roles in maintaining chloroplast Mg^2+^ homeostasis.

## 1 Introduction

Magnesium (Mg^2+^) is an abundant and essential mineral nutrient for all living organisms. In plants, Mg^2+^ critically contributes to the process of photosynthesis since it is required as the central ion of the chlorophyll (Chl) molecule and as an activator of RuBisCO as well as for thylakoid stacking and counterbalancing of the H^+^ gradient across the thylakoid membrane (Szabò and Spetea, 2017; Tang and Luan, 2017). A significant proportion (15-35%) of the total Mg^2+^ content in plants is allocated to the chloroplast (Chen et al., 2018). In *Arabidopsis thaliana* (hereafter Arabidopsis), Mg^2+^ is taken up from the soil by the roots, loaded into the xylem, and transported throughout the shoots into the leaf chloroplasts (Hermans et al., 2013). Due to its charge, Mg^2+^ cannot move freely across membranes, and transport is tightly controlled by specialized transport proteins.

Even though no thylakoid-located Mg^2+^ transporter has been identified so far, two distinct families of proteins are known to transport Mg^2+^ across the inner envelope membrane. MGT10 was localized to the chloroplast envelope (Drummond et al., 2006), proven to transport Mg^2+^, and to play an essential role in chloroplast development and photosynthesis (Liang et al., 2017; Sun et al., 2017). MGT10 belongs to a major family of magnesium transporters in plants (MGTs) that are related to the well-characterized family of bacterial CorA-type Mg^2+^ ion channels (Li et al., 2001; Lunin et al., 2006; Guskov et al., 2012). Most recently, two magnesium release transporters, MGR8 and MGR9, from a distant clade of cyclin M (CNNMs) from yeast and humans, were localized to the chloroplast inner envelope (Zhang et al., 2022). MGR8 and MGR9 share 78% amino acid sequence identity and their Mg^2+^ uptake activity was demonstrated by functional complementation of a *Salmonella typhimurium* mutant (Zhang et al., 2022). Both families of Mg^2+^ transporters play an essential role for the plant since the single knockout of *MGR10* and the double knockout of *MGR8* and *MGR9* result in impaired chloroplast development (Sun et al., 2017; Zhang et al., 2022). At present, the individual roles of these transporters remain largely unknown.

Mg^2+^ is also essential for photosynthesis and growth in algae, and although there are homologs in the genomes of green algae, no Mg^2+^ transporter has been characterized so far (Marchand et al., 2018). In this study, we aimed to assess the role of MGR8, MGR9, and MGT10 in maintaining magnesium homeostasis in the chloroplast of Arabidopsis and if their role is conserved in the model green alga *Chlamydomonas reinhardtii* (hereafter Chlamydomonas). To reach this aim, we analyzed and compared gene expression, Mg^2+^ content, proton motive force (PMF) size and partitioning, non-photochemical quenching (NPQ), chloroplast ultrastructure, and biomass in wild type, corresponding single and double mutants when cultivated hydroponically in standard as well as low and high Mg^2+^ conditions. Using functional complementation assays in *Escherichia coli* (*E. coli*), we show that MGR8 and MGR9 are capable of mediating Mg^2+^ transport although with different affinities. In Arabidopsis leaves, together with MGT10, they regulate photosynthetic electron transport and photoprotection in response to excess light. Chlamydomonas does not have chloroplast MGRs, but the MGT10 homolog (MRS4) is required for photosynthesis and cell growth.

## 2 Results

### 2.1 MGR8 and MGR9 resemble CorC-like transporters, whereas MGT10 is a CorA-like channel

**Figure 1:**
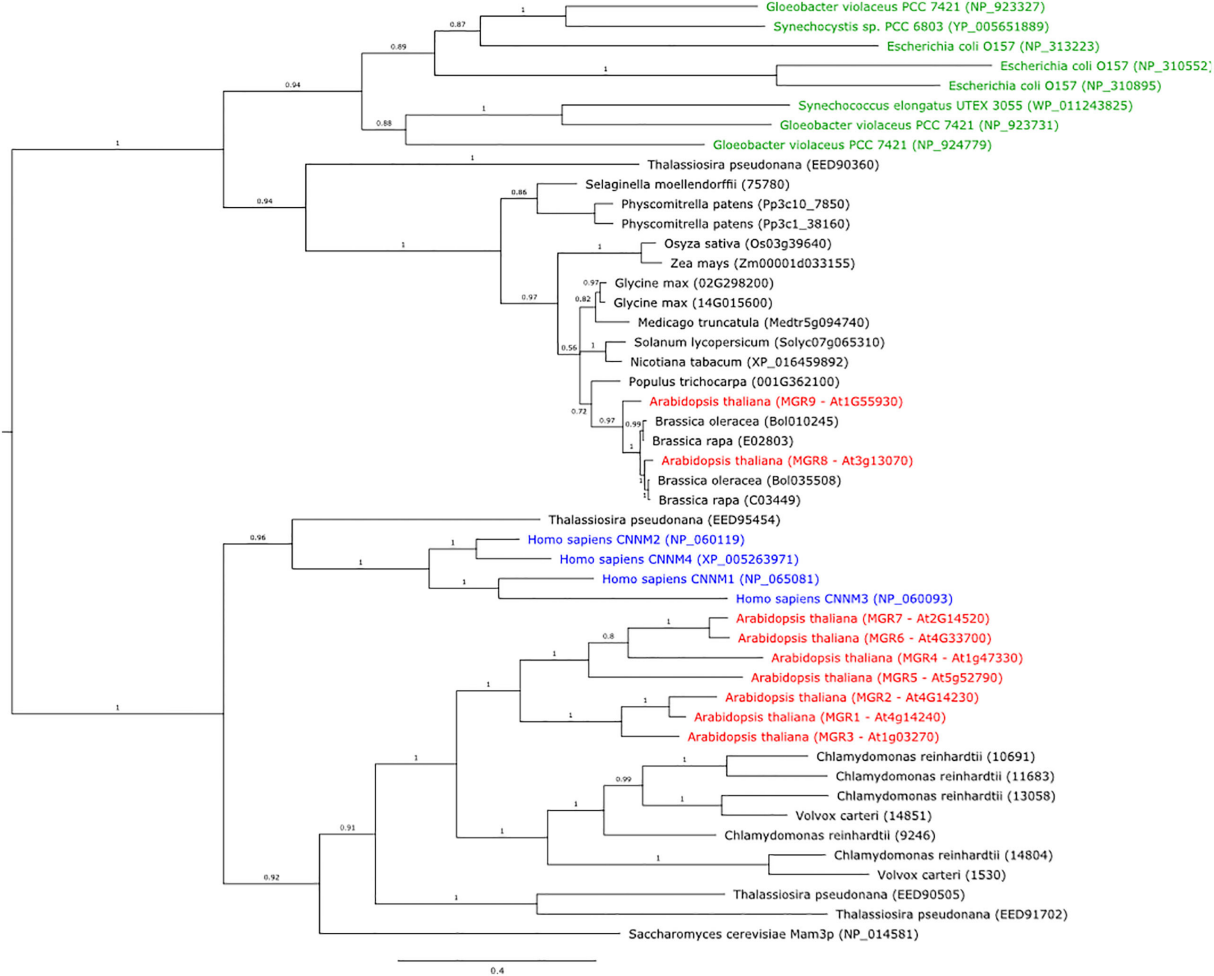
Phylogenetic tree of protein sequences from plants, bacteria, green algae, diatoms, yeast, and humans, that are homologs of the Arabidopsis MGR8 and MGR9. The tree was constructed using MrBayes v3.2.6. Bacteria are indicated in green text, the human CNNM1–4 homologs in blue, and all Arabidopsis MGRs in red. Numbers above branches indicate posterior probability values and the expected number of changes per site along the branches is indicated by the scale bar.

To assess the evolutionary relationships among MGT10, MGR8, and MGR9, we compared their protein sequences with those of several well-characterized magnesium transporters. Phylogenetic analyses showed that MGT10 shared the closest evolutionary history with the *E. coli* CorA, whereas MGR8 and MGR9 clustered together with the CorC proteins of *Thermus parvatiensis* and *E. coli* (**Supplementary Figure S1A**). The magnesium transport function of CorA protein family members depends on the Gly-Met-Asn (GMN) motif located in the extracellular loop of the channel (Guskov et al., 2012; Ishijima et al., 2021). This motif could be found in the MGT10 sequence, whereas it was absent in MGR8 and MGR9 sequences (**Supplementary Figure S1B**). Previous work reported that MGR8 and MGR9 belong to a separate clade of the plant MGR family, which is most distant from CNNMs (Tang et al., 2022; Zhang et al., 2022). Our phylogenetic analyses confirmed that MGR8 and MGR9 cluster together in a MGR sub-family (**Figure 1**). Interestingly, this cluster does not include CNNMs and MGR1–7 and does include the bacterial Mg^2+^ transporter CorC (**Figure 1**, **Supplementary Figure S1A**). Structurally, CNNMs consist of an N-terminal extracellular domain, a transmembrane domain of unknown function (DUF21), a large cytosolic region containing a cystathionine-synthase (CBS) pair domain, and a putative cytosolic cyclic nucleotide–binding homology (CNBH) domain at the C-terminus. Our sequence alignment shows that while MGR8 and MGT9 also harbor the DUF21 and the CBS-pair domain, the C-terminus is distinct from the CNBH domain of CNNMs and shares a considerable number of identical amino acids with the C-terminal CorC-HlyC domain of bacterial CorC (**Supplementary Figure S1B**). Members of the CorC Mg^2+^ transporter family were shown to be Na^+^ dependent since depletion of Na^+^ resulted in loss of their Mg^2+^ transport activity (Yamazaki et al., 2013; Jin et al., 2022). The Asparagine residue Asn94 in the transmembrane domain of the CorC protein from *Thermus parvatiensis* was identified as important for Na^+^ sensitivity (Huang et al., 2021). Interestingly, both MGR8 and MGR9 also contain this residue in their transmembrane DUF21 domain (**Supplementary Figure S1B**), suggesting possible coordination of transport between Mg^2+^ and Na^+^ across the chloroplast inner envelope. Taken together, these data indicate that MGR8 and MGR9 may function as CorC-like transporters, whereas MGT10 shares the closest evolutionary history with the CorA channel.

Phylogenetic analysis of MGR8 and MGR9 homologs revealed that both proteins are distributed amongst land plants, algae, and cyanobacteria. Within the plant genus *Brassicaceae*, MGR8 and MGR9 proteins form two well-supported clades and all investigated species code for 2–3 copies each (**Supplementary Figure S2**). However, species from *Brassica*, *Eruca*, *Crambe*, and *Sinapis*, only carry the MGR8-like protein, albeit in multiple distinct copies, resulting from gene duplication. There are 1–2 copies of MGR8- and MGR9-related proteins in other plant species and cyanobacteria species like *Synechocystis* sp., *Synechoccocus elongatus*, and *Gloeobacter violaceus* (**Figure 1**). In diatoms (e.g., *Thalassiosira pseudonana*), we could find homologs for CNNMs and MGR1–7 but not for MGR8 and MGR9. These findings indicate that MGR8 and MGR9 share evolutionary histories but are not evenly distributed in nature.

To assess if the Mg^2+^ transport protein sequences of Arabidopsis MGR8, MGR9, and MGT10 are conserved in green algae, we have searched for homologs in the unicellular *Chlamydomonas reinhardtii* and the multicellular *Volvox carteri*. While there was no AtMGR8 or AtMGR9 homolog, we identified one MRS4 sequence in each green algae species as close homologs of AtMGT10 (**Supplementary Figure 3**). MRS4 shares the highest sequence identity with MGT10 in the N-terminal long loop and in the transmembrane region (**Supplementary Figure S4**). In addition, the protein sequences of both CrMRS4 and VcMRS4 contain the characteristic GMN motif.

### 2.2 MGR8 and MGR9 transport Mg^2+^ in *E. coli* by a different mechanism than MGT10 and CNNMs

**Figure 2:**
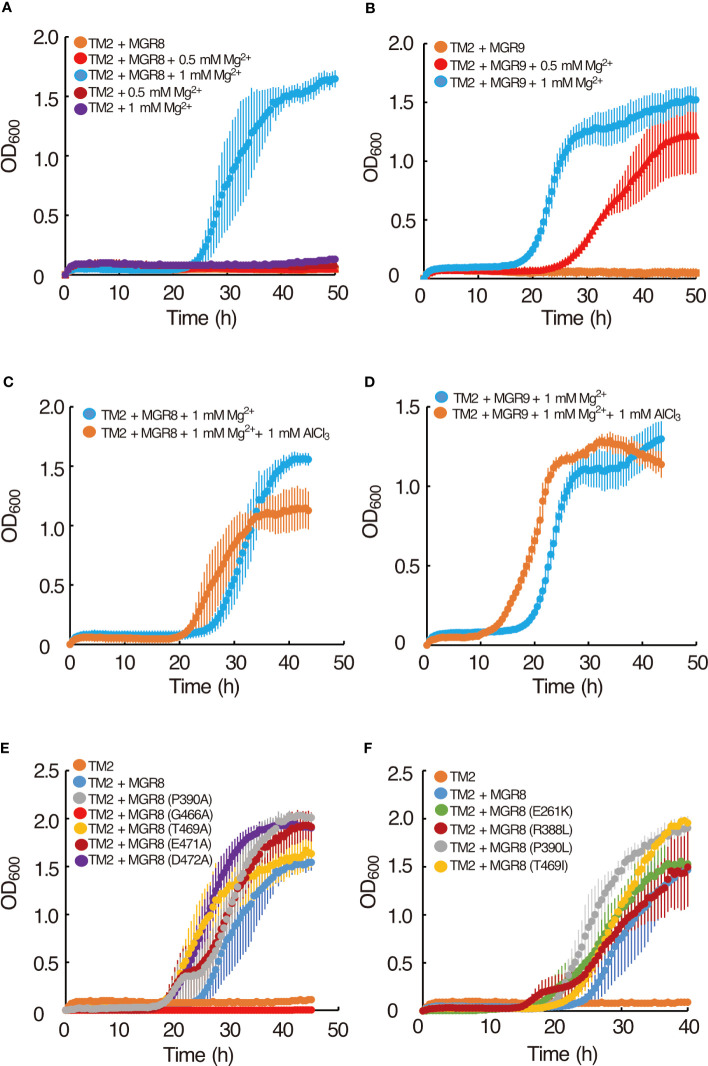
Complementation of the *E. coli* TM2 Mg^2+^ auxotrophy with *MGR8* and *MGR9* and Al^3+^ inhibition. **(A)** Growth curves of TM2 transformed with the pTV118N vector containing *MGR8* cDNA and with the empty vector. **(B)** Growth curves of TM2 transformed with the plasmid containing *MGR9* cDNA. Cells were grown at 37°C on LB medium supplemented with different concentrations of MgSO_4_. LB medium without added MgSO_4_ contained 0.17 mM Mg^2+^ (Ishijima et al., 2015). **(C, D)** Effect of Al^3+^ on the growth curves of TM2 transformed with the plasmid containing *MGR8* **(C)** and *MGR9* **(D)** cDNA. Cells were grown at 37°C on LB medium supplemented with 1 mM MgSO_4_. AlCl_3_ was added at 0 mM and 1 mM concentration. **(E, F)** Growth curves of TM2 transformed with the plasmid containing *MGR8* wild type, P390A, G466A, T469A, E471A, and D472A **(E)**, E261K, R388L, P390L, and T469I **(F)** mutant cDNA and with the empty pTV118N vector. Cells were grown at 37°C on LB medium supplemented with 1 mM MgSO_4_. The OD_600_ was measured every 0.5 h. Data are average values of three or more independent experiments, and bars indicate means ± S.E.M.

Using heterologous expression in the Mg^2+^ uptake-deficient *E. coli* strain TM2 (Δ*corA* Δ*mgtA* Δ*yhiD*), we assessed the magnesium transport activity of MGR8 and MGR9 and compared it with that of MGT10. Under standard conditions, the growth of TM2 requires the addition of at least 10 mM Mg^2+^ to the LB medium (Ishijima et al., 2015). TM2 cells expressing either *MGR8* or *MGR9* cDNA grew optimally in LB medium supplemented with 1 mM Mg^2+^ but not in the absence of added Mg^2+^, while the cells with an empty vector did not grow in either absence or presence of up to 1 mM Mg^2+^ (**Figures 2A, B**). Interestingly, TM2 cells expressing *MGR9* could grow well in LB medium supplemented with 0.5 mM Mg^2+^, while *MGR8*-expressing cells failed to grow (**Figures 2A, B**). In addition, when 0.8 mM or less Mg^2+^ was added, the growth of cells expressing *MGR8* but not of cells expressing *MGR9* was reduced (**Supplementary Figures S5A, B**). At 10 mM Mg^2+^, the cells expressing *MGR8* grew much faster and better than the *MGR9*-expressing cells and the cells with an empty vector (**Supplementary Figure S5C**). As previously reported by Ishijima and colleagues (2015; 2021), we observed that TM2 cells expressing *MGT10* could readily grow in LB medium without Mg^2+^ supplementation (**Supplementary Figure S5D**). These results indicate that MGR8, MGR9, and MGT10 functionally complement the Mg^2+^ auxotrophy of the TM2 cells and that the expressed proteins are capable of transporting Mg^2+^ without any additional protein partners. The ability of TM2 cells expressing *MGR9* to grow at lower external Mg^2+^ concentration than the cells expressing *MGR8* indicates a broader concentration range at which MGR9 may be active.

We found that AlCl_3_ inhibited the growth of TM2 cells expressing *MGT10* (**Supplementary Figure S5D**) as previously reported (Ishijima et al., 2015), likely due to Al^3+^ uptake into the cells (Ishijima et al., 2018). In contrast, no growth inhibition of TM2 cells expressing either *MGR8* or *MGR9* was observed with 1 mM AlCl_3_ in combination with 1 mM Mg^2+^ (**Figures 2C, D**). These results indicate that the Mg^2+^ transport activity of MGR8 and MGR9 is not inhibited by Al^3+^ and that they do not transport Al^3+^ into the *E. coli* cells under these conditions. Taken together, we propose that MGR8 and MGR9 transport Mg^2+^ by a different mechanism than MGT10.

Human CNNMs contain evolutionarily conserved residues, whose mutations cause hypomagnesemia and associated congenital diseases (Stuiver et al., 2011; Giménez-Mascarell et al., 2019; Chen Y.S. et al., 2020). Disease-causing mutations include E357K from the DUF21 domain of CNNM2, R407L, P409L, and T495I from the ATP-binding site within the CBS domain of CNNM4 (Chen Y.S. et al., 2020), demonstrating the importance of these four residues for CNNM transport activity. In addition, the residues corresponding to G466, E471, and D472 of MGR8 are conserved near the end of the CBS domain in the CNNM family (Zhang et al., 2022). To test whether these seven residues (**Supplementary Figure S1B**) are important for the Mg^2+^ transport activity of MGR8, we introduced point mutations (E261K, R388L, P390A, P390L, G466A, T469A, T469I, E471A, and D472A) and expressed the constructs in TM2 cells. The TM2 cells expressing the MGR8 G466A mutant did not grow in LB medium supplemented with 1 mM Mg^2+^ (**Figure 2E**). All other mutants grew similarly to the cells expressing the wild type MGR8 (**Figures 2E, F**). These results indicate that, among the mutated residues conserved in CNNMs, only G466 in the CBS domain is critical for the Mg^2+^ transport activity of MGR8. Based on these findings and the notion that all mutated residues are also conserved in MGR9 (**Supplementary Figure S1B**), we postulate that MGR8 and MGR9 transport Mg^2+^ by a different mechanism than CNNMs.

### 2.3 *MGR9* gene expression is upregulated at low and high Mg^2+^ concentrations

**Figure 3:**
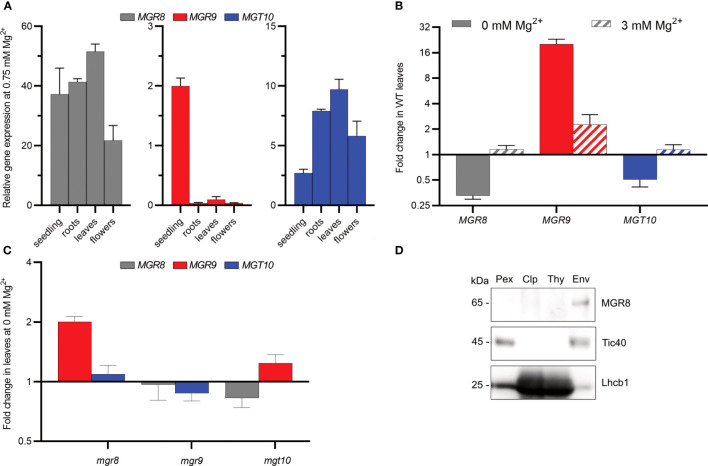
Expression pattern and chloroplast localization in Arabidopsis. **(A)** Relative expression in 2-week-old seedlings and roots, leaves, and flowers of 6-week-old wild type plants grown hydroponically at 0.75 mM Mg^2+^ was determined using quantitative RT-PCR. *ACTIN8* and *PEX4* were used as internal standards. **(B, C)** Wild type (WT) plants **(B)** and mutants **(C)** were grown first for two weeks at 0.75 mM Mg^2+^ and then for three to four weeks at either 0 or 3 mM Mg^2+^. The plotted data represent expression fold change relative to the expression at 0.75 mM Mg^2+^ in the same genotype. Where two independent lines were available, the obtained data are presented as averages. The scale on the Y-axis in **(B, C)** is log_2_, whereas the fold change values are non-log_2_-transformed. The data presented in **(A–C)** are means ± S.E.M (*n* = 4 plants). **(D)** Total protein extracts (Pex), Chloroplasts (Clp), thylakoid (Thy), and envelope (Env) membranes were prepared from wild type leaves. Localization of MGR8 in chloroplast and subfractions was performed by immunoblotting with an MGR8-peptide-specific antibody. Purity of fractions was confirmed using antibodies against marker proteins for the respective compartment: inner envelope translocon complex Tic40 protein and the light harvesting Chl *a/b* binding thylakoid protein Lhcb1. Uncropped version of the immunoblots is shown in **Supplementary Figure S8**.

The tissue-specific expression pattern of the *MGT10* gene was previously analysed by GUS-staining (Drummond et al., 2006; Sun et al., 2017). Recently, the more sensitive quantitative RT-PCR method was used to analyze the expression of *MGR8* and *MGR9* genes (Zhang et al., 2022). In our quantitative RT-PCR analysis we included all three transporter genes and investigated their tissue expression pattern in Arabidopsis plants grown at 0.75 mM Mg^2+^ (standard conditions). *MGR8* exhibited high expression in all vegetative organs and lower expression in flowers (**Figure 3A**). *MGT10* was highest expressed in mature organs, in contrast to *MGR9* which was predominantly expressed in seedlings. Notably, expression levels of *MGR8* and *MGT10* genes were highest in leaves.

Considering the observed activity of MGR9 in TM2 cells under low Mg^2+^ conditions, we postulated the possible involvement of MGR9 in Arabidopsis growth at such levels. Accordingly, we evaluated gene expression in response to three different Mg^2+^ concentrations. Plants were grown for two weeks at 0.75 mM Mg^2+^ and then transferred for 3–4 weeks at either no (0 mM), standard (0.75 mM), or high (3 mM) Mg^2+^. At 0 mM Mg^2+^, expression of the *MGR9* gene in leaves was upregulated 20-fold, while expression of *MGR8* and *MGT10* was downregulated as compared to standard conditions (**Figure 3B**). *MGR9* expression was also upregulated at 3 mM Mg^2+^ (2-fold), while the expression of the other two genes was unaltered relative to 0.75 mM Mg^2+^. These results indicate that *MGR8* and *MGT10* are the mainly expressed Mg^2+^ transporters in the leaves of Arabidopsis plants grown at standard Mg^2+^ concentration, whereas *MGR9* expression is elevated in conditions of deficiency or excess Mg^2+^.

To further investigate the gene expression patterns of the three magnesium transporters under Mg^2+^-deficient conditions, we obtained two independent homozygous T-DNA insertion knockout mutants for *MGR8* (*mgr8-1* and *mgr8-2*) and *MGR9* (*mgr9-1* and *mgr9-2*), and a heterozygous knockdown mutant for *MGT10* (*mgt10*) (**Supplementary Figure S6**). We speculate that these mutant plants might compensate for the loss of one transporter by upregulating the expression of the other magnesium transporter genes. The expression of *MGR9* was similar in *mgr8* and *mgt10* mutants grown hydroponically at 0.75 mM Mg^2+^, whereas *MGR8* was more expressed in *mgt10* than in *mgr9* (**Supplementary Figure S7**). When grown at 0 mM Mg^2+^, *MGR9* was upregulated 2-fold in *mgr8* and slightly upregulated in *mgt10*, whereas *MGT10* and *MGR8* expression was downregulated or unaltered in *mgr9* and *mgt10* mutants relative to 0.75 mM Mg^2+^ (**Figure 3C**). These results strengthen the evidence for a role of MGR9 in Mg^2+^-deficient conditions.

Knowing a protein’s subcellular localization is a pivotal element in unravelling its functional role within the cell. Sun and colleagues (2017) localized MGT10 to the chloroplast envelope by western blot analysis. Recently, MGR8 and MGR9 were also localized to the chloroplast envelope by using a GFP-fluorescence approach (Zhang et al., 2022). In our study, we raised an antibody against an MGR8-specific C-terminal peptide (**Supplementary Figure S1B**). The generated antibody detected a band at a relative molecular weight (*Mr*) of 65 kDa in total protein extracts from wild type (WT) leaves and in mutant lines *mgr9-1* and *mgr9-2*, but not in *mgr8-1* and *mgr8-2*. The *Mr* is in good agreement with the theoretical MW of 65.65 kDa for the protein lacking the chloroplast transit peptide (amino acids 1-71, **Supplementary Figure S1B**). Immunoblot analyses of purified chloroplasts membrane subfractions, thylakoid- and envelope membranes confirmed an envelope location of the MGR8 protein (**Figure 3D** and **Supplementary Figure S8**).

### 2.4 MGR8, MGR9, and MGT10 are required for regular thylakoid stacking in Arabidopsis

**Figure 4:**
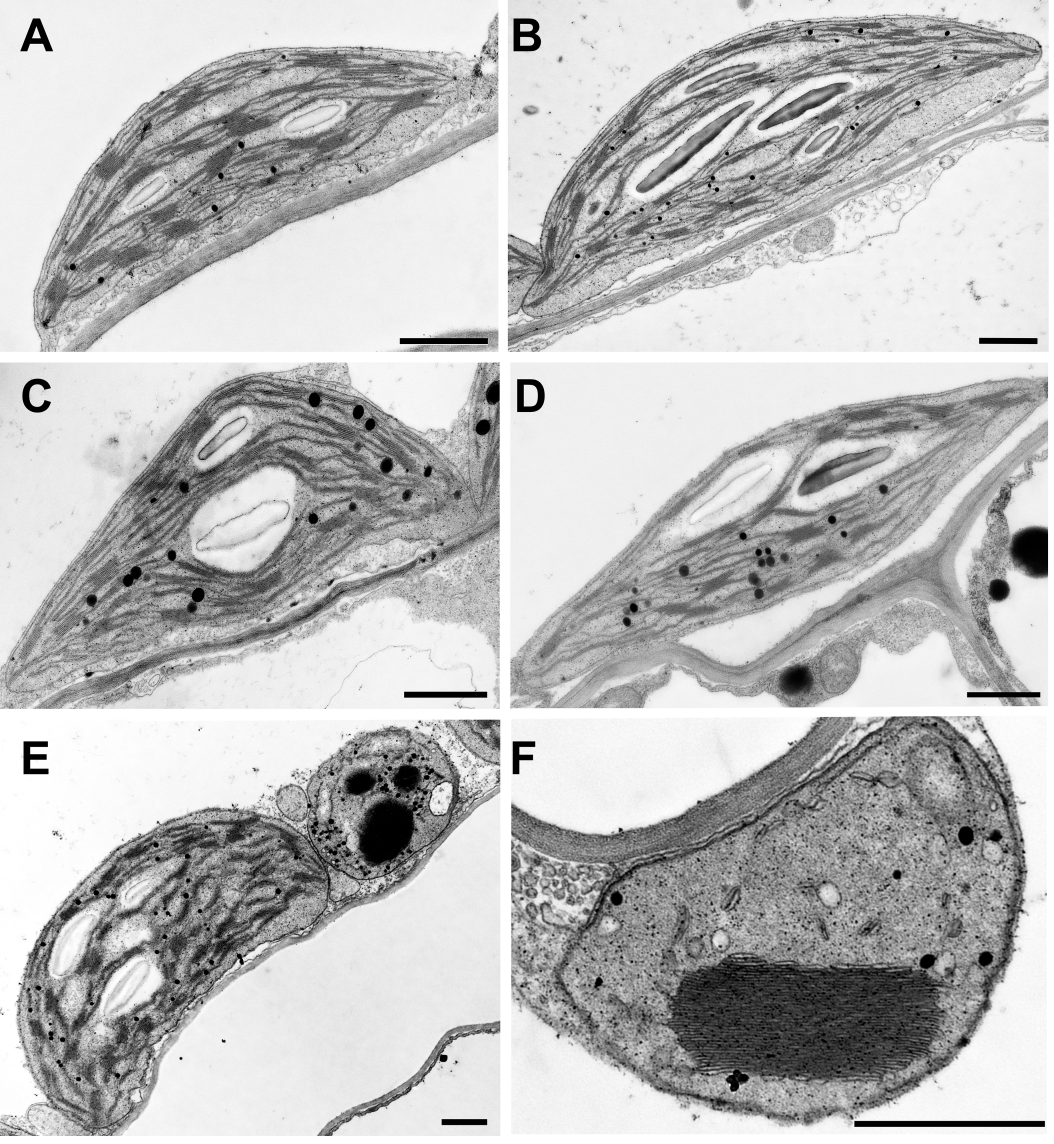
Chloroplast ultrastructure in Arabidopsis. Wild type plants and mutants were grown in standard Mg^2+^ conditions for six weeks. Representative TEM photos of chloroplasts are shown. **(A)** Wild-type, **(B)** *mgr8-2*, **(C)** *mgr9-1*, **(D)** *mgt10* green interveinal region, **(E, F)** *mgt10* chlorotic, yellow vein region. **(E)** Mesophyll cell with normal chloroplast and peculiar plastid. **(F)** peculiar plastid with macro-granum, vesicles, and no stroma thylakoids typical for bundle sheath cells. Scale bar: 1 μm.

Thylakoid stacking, also referred to as overall grana size, is stabilized by Mg^2+^ ions. We, therefore, examined the chloroplast ultrastructure and thylakoid stacking in three single mutant lines using transmission electron microscopy (TEM). Regular WT-like chloroplast and thylakoid morphology were observed in 6-week-old *mgr8* and *mgr9* mutants (**Figures 4A–C**). Interestingly, it was reported that the homozygous double mutant *mgr8mgr9* had impaired chloroplast development (Zhang et al., 2022). This double mutant had no thylakoid stacks in two-week-old plants and short and inflated thylakoid stacks in 3-week-old plants.

The *mgt10* mutant had a peculiar leaf phenotype with yellow vein leaves (**Supplementary Figure S9**), in agreement with previously published results (Liang et al., 2017; Sun et al., 2017). The green interveinal regions contained chloroplasts with regular grana (**Figure 4D**), whereas the yellow vein regions contained normal chloroplasts with regular grana and peculiar plastids with macro-grana (**Figures 4E, F**). Macro-grana are unusually wide grana consisting of a high number of stacked thylakoid lamellae and are associated with an overall underdeveloped stroma thylakoid membrane system. Macro-grana were especially abundant in the bundle sheath cells, although they could be also observed in palisade and spongy parenchyma cells sampled from the vein region. Taken together, these results in combination with the results from Zhang and colleagues (2022) suggest that both families are required for regular thylakoid stacking and chloroplast development. However, the opposite pattern in grana size, i.e., smaller in *mgr8mgr9* (Zhang et al., 2022) and larger in the peculiar plastids observed in *mgt10* (**Figure 4F**, Sun et al., 2017), implies differential roles of the two families of transporters.

### 2.5 MGR8, MGR9, and MGT10 participate in building the pH gradient required for photoprotection in fluctuating light

To understand the role of MGR8, MGR9, and MGT10 in photosynthetic reactions in the thylakoid membrane, we grew plants at three different concentrations of Mg^2+^ (0, 0.75, and 3 mM) and measured the slow kinetics of chlorophyll fluorescence induction in fluctuating light. In addition to the single mutants, we investigated the impact of a double mutant of the two most expressed transporters on photosynthetic performance. We crossed the *mgr8-2* and *mgt10* mutant lines, resulting in the *mgt10mgr8-2* double mutant. All mutant lines grew like WT and displayed similar shoot and root weight at all three Mg^2+^ concentrations (**Supplementary Figure S9**). We first determined the maximum quantum yield of photosystem II (PSII) photochemistry (F_v_/F_m_). Our results show that the F_v_/F_m_ yield was slightly but significantly lower in both *mgr9* lines and *mgt10* at 0 mM Mg^2+^, indicating a reduced maximum photosynthetic efficiency in terms of electron transport (**Supplementary Table S1**).

**Figure 5:**
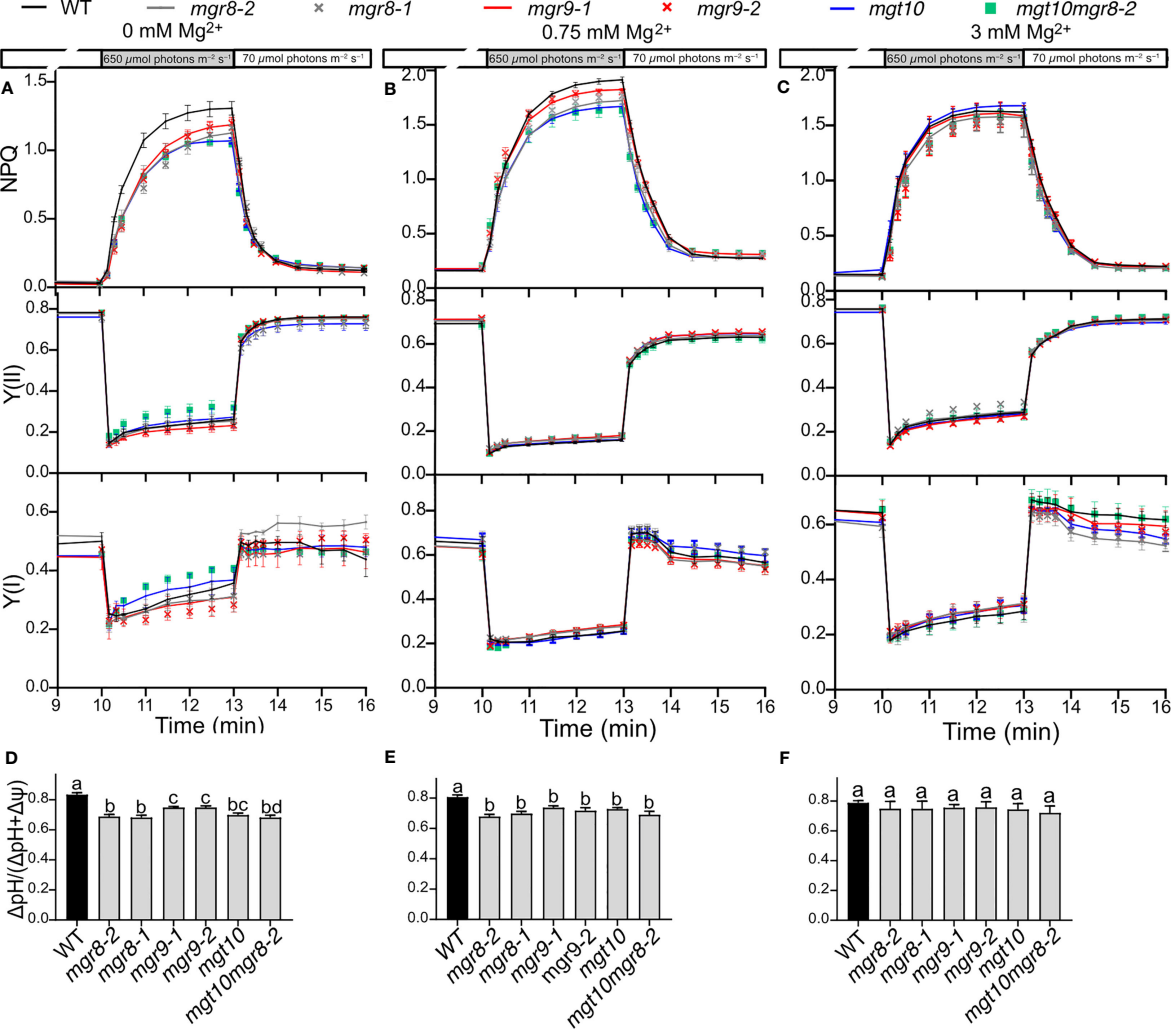
Dynamics of photosynthesis and photoprotection in fluctuating light. Wild type (WT) plants and mutants were grown hydroponically first for two weeks at 0.75 mM Mg^2+^ and then for four to five weeks at the indicated Mg^2+^ concentrations. Plants were dark adapted for 20 min, illuminated for 10 min with low light, then for 3 min with high light, and then again for 3 min in low light. Chl fluorescence and electrochromic shift were recorded with Dual-PAM-100. The plots in **(A–C)** show non-photochemical quenching (NPQ), PSII, and PSI quantum yields (Y(II) and (YI)). The plots in **(D–F)** show the partitioning of the proton motive force to ΔpH as determined from ECS measurements at the end of transition from low to high light. The plotted data are means ± S.E.M. (n = 4-7 plants). Different letters indicate statistically significant differences among the genotypes according to Tukey one-way ANOVA (*P* < 0.05).

In plants grown at 0 and 0.75 mM Mg^2+^, on transition from low to high light, NPQ was induced slower in all mutants as compared to WT (**Figures 5A, B**). The steady-state NPQ was the lowest in *mgt10* and *mgt10mgr8-2*, intermediate in *mgr8*, and the least affected in the *mgr9* mutants. Following the transition from high to low light, NPQ relaxed similarly in all genotypes. The electron transport through both photosystems, indicated by Y(II) and Y(I), was like WT in all mutants and conditions (**Figures 5A, B**). In parallel experiments, we recorded electrochromic shift (ECS) kinetics at the end of each low-to-high light and high-to-low light transition to estimate the total PMF and its partitioning. The PMF size was alike WT at the end of each transition (**Supplementary Figure S10A**). The ΔpH, also known as the H^+^ concentration gradient, in transition from low to high light significantly decreased in all mutants grown at 0 and 0.75 mM Mg^2+^ (**Figures 5D, E**), and was like WT in transition from high to low light (**Supplementary Figure S10B**). These data indicate that MGR8, MGR9, and MGT10 are involved in building up the pH gradient to rapidly activate NPQ without largely affecting the electron transport through photosystems and overall PMF size. Plants grown at 3 mM Mg^2+^ did not differ in any of the studied parameters (**Figures 5C, F**, **Supplementary Figures S10A, B**).

**Figure 6:**
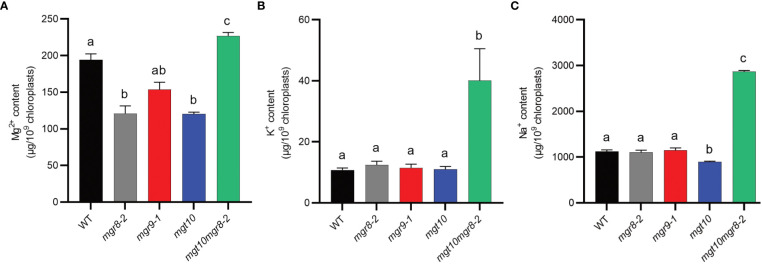
Mineral content of Arabidopsis chloroplasts. Intact chloroplasts were prepared from shoots of plants grown at 0.75 mM Mg^2+^ and their mineral content was determined using ICP-MS. **(A)** Mg^2+^, **(B)** K^+^, and **(C)** Na^+^ content of Arabidopsis chloroplasts. The presented data are expressed as means ± S.E.M. (*n* = 3 chloroplast preparations). Different letters indicate statistically significant differences among the genotypes according to Tukey one-way ANOVA (*P* < 0.05).

To test whether the observed reduced NPQ and altered thylakoid ultrastructure could be a result of altered magnesium homeostasis, we measured the mineral content in shoots and isolated chloroplasts using inductively coupled plasma optical emission spectrometry (ICP-OES) and inductively coupled plasma mass spectrometry (ICP-MS), respectively. When grown in the absence of Mg^2+^, all mutants had a slightly but significantly higher Mg^2+^ content in the shoots compared to WT (**Supplementary Figure S11**). At standard Mg^2+^ concentration (0.75 mM), shoots of all single mutants had less whereas the double mutant *mgt10mgr8-2* had a WT-like Mg^2+^ content. At 3 mM Mg^2+^, all mutant shoots had WT levels of Mg^2+^ except for *mgt10* which had a significantly higher content. In chloroplasts isolated from plants grown at standard Mg^2+^ concentration, *mgr8-2* and *mgt10* had a Mg^2+^ content reduced by 38%, *mgr9* by 21%, whereas *mgt10mgr8-2* displayed a 17% increase relative to WT (**Figure 6A**). The chloroplast Na^+^ and K^+^ contents in most single mutant lines were similar to those in the WT (**Figures 6B, C**). Nevertheless, *mgt10mgr8-2* contained significantly higher levels of both Na^+^ and K^+^, suggesting that the double loss of MGR10 and MGR8 also impacts other ion transporters.

To investigate whether the observed differences in the chloroplast Mg^2+^ content have impacted RuBisCO activity in CO_2_ fixation, we have measured net photosynthesis at the growth light and at a higher light intensity. As shown in **Supplementary Figure S12**, this activity was not significantly different among the genotypes at neither light intensity, in line with the WT-like growth and biomass data (**Supplementary Figure S9**).

### 2.6 The Chlamydomonas MRS4 transporter is required for photosynthesis and cell growth

**Figure 7:**
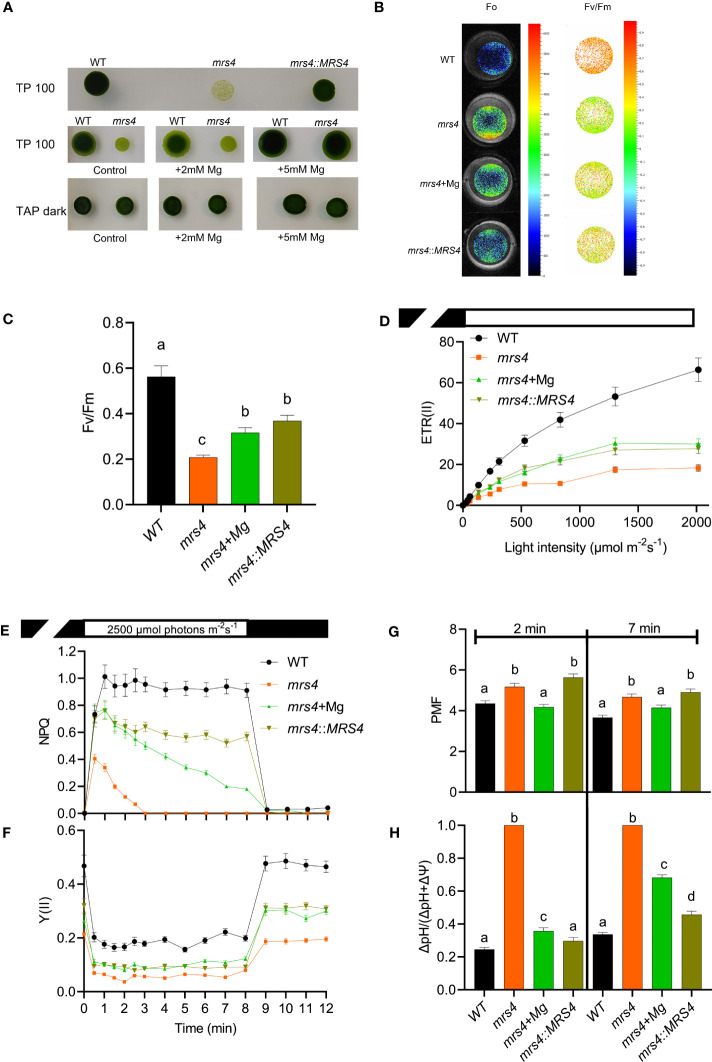
Chlamydomonas *mrs4* experiences light stress. Wild type (WT), *mrs4*, and complemented *mrs4:MRS4* were grown in the light (100 μmol photons m^−2^ s^−1^) on TP medium (TP100) or in darkness on TAP (TAP dark), and where indicated supplemented with 5 mM Mg^2+^. **(A)** Spot tests on agar plates showing that complementation with *VcMRS4* and Mg^2+^ supplementation improve the growth of the mutant. **(B, C)** Chl fluorescence imaging using FluorCam shows significantly reduced maximum quantum yield of PSII photochemistry (F_v_/F_m_) in *mrs4* due to enhanced minimum fluorescence (F_0_). Complementation with *VcMRS4* and Mg^2+^ supplementation improved the photosynthetic efficiency of the mutant. Chl fluorescence and electrochromic shift were recorded with a Dual-Pam-100. **(D)** Rapid light response curves of electron transport rates of PSII (ETR(II)). **(E, F)** Slow kinetics of Chl fluorescence induction during 8 min of illumination followed by 4 min in darkness. The data show reduced ETR(II), NPQ, and Y(II) in *mrs4* and improvement by complementation with *VcMRS4* and Mg^2+^ supplementation of cells grown in TP100. **(G, H)** The total proton motive force (PMF) and partitioning to ΔpH were determined from ECS decay kinetics in darkness of cells grown in TAP dark and pre-exposed at 660 μmol photons m^−2^ s^−1^ for 2 or 7 min. The data in **(C–G)** are means ± S.E.M. (*n* = 3 replicates). Different letters indicate significant differences among the genotypes according to Tukey one-way ANOVA (*P* < 0.05). The PMF in the *mrs4* mutant consists of 100% ΔpH, indicating that the cells experience high light stress.

We identified CrMRS4 and VcMRS4 proteins as close homologs of AtMGT10 (**Supplementary Figures S3**, **S4**). To explore the function of MRS4, we characterized a Chlamydomonas knock-out mutant from the CLiP library (*mrs4*) and complemented this mutant with the *VcMRS4* gene (*mrs4*::*MRS4*) (**Supplementary Figure S13**). The knockout mutant grew poorly in light on minimum TP medium, but supplementation with Mg^2+^ or complementation with *VcMRS4* considerably improved its growth (**Figure 7A**). Growth of *mrs4* in TAP medium in darkness did not impact growth (**Figure 7A**), indicating that MRS4 is involved in autotrophic growth.

To further investigate the cause of the reduced growth of *mrs4*, we examined various parameters of photosynthetic reactions in the thylakoid membrane. The knockout mutant *mrs4* had a significantly reduced F_v_/F_m_ relative to WT due to a higher F_0_. These results indicate that *mrs4* had a higher proportion of closed PSII centers already in the dark-adapted state (**Figures 7B, C**). In addition, *mrs4* had reduced electron transport rates throughout the range of tested light intensities, indicating enhanced light sensitivity (**Figure 7D**). When exposed to high light (2500 µmol photons m^-2^ s^-1^), the knockout mutant was able to induce NPQ in the first minute of illumination, but it decreased to 0 after 3 min. The NPQ in the supplemented and complemented lines were higher than in *mrs4* and more stable over time, but still lower than WT (**Figure 7E**). In addition, the PSII activity, indicated by Y(II), was lower in all mutants (**Figure 7F**). The total PMF across the thylakoid membrane was slightly but significantly higher in *mrs4* and in *mrs4*::*MRS4* at two time points in high light (660 µmol photons m^-2^ s^-1^), and like WT in the supplemented line (**Figure 7G**). The *mrs4* mutant PMF was dominated by ΔpH, whereas the complemented line and the Mg^2+^ supplemented mutant had intermediate ΔpH levels between WT and mutant (**Figure 7H**, **Supplementary Figure S14**). The observed high ΔpH in the *mrs4* mutant is likely to cause lower PSII activity due to photoinhibition. Notably, despite the high ΔpH, the NPQ levels in the mutant were low, indicating a reduced capacity for photoprotection (**Figures 7D, F**). Taken together, these results suggest that the *mrs4* mutant has an enhanced sensitivity to light due to photoinhibition and malfunctioning of the PSII complex.

To test if the observed lower PSII activity is linked to an altered thylakoid structure, WT and *mrs4* morphology were analyzed using TEM. Chlamydomonas cultures grown in TAP medium in darkness revealed no major differences between WT and *mrs4* (**Supplementary Figure S15A**). In addition, a similar overall cell ultrastructure was observed in WT and the *mrs4* mutant after 3 h or 4 days of illumination at 100 µmol photons m^-2^ s^-1^ on minimal TP medium. However, inside the chloroplast, the *mrs4* thylakoids were more stacked and consisted of more layers (**Supplementary Figures S15A, B**).

To investigate if the decrease in photoprotective capacity, increased ΔpH and altered grana stacking could be a result of altered mineral content in the cell, we measured the Mg^2+^/Na^+^, K^+^/Na^+^, and Ca^2+^/Na^+^ ratios of *mrs4* and WT using ICP-MS. There were no significant differences in the mineral content ratios for cells grown on TAP in darkness (**Supplementary Figure S16**). However, we found that *mrs4* cells grown at 100 µmol photons m^-2^ s^-1^ in minimal TP medium had significantly elevated Mg^2+^/Na^+^ and Ca^2+^/Na^+^ ratios as compared to WT (**Supplementary Figure S16**). These results indicate that *mrs4* accumulated higher levels of Mg^2+^ and Ca^2+^ inside the cell. An excess of Mg^2+^ could explain the increased grana size, as it was proposed for MGT10 in Arabidopsis (Sun et al., 2017). Taken together with the observed altered thylakoid ultrastructure and associated reactions, our data suggests that MRS4 ensures an optimal Mg^2+^ concentration in the chloroplast for photosynthesis and cell growth.

## 3 Discussion

Plants require Mg^2+^ inside the chloroplast for chlorophyll synthesis, RuBisCO enzyme activation, and thylakoid stacking. As a result, this ion plays a pivotal role in both light-dependent and light-independent photosynthetic reactions. The inner envelope of the chloroplast contains three different Mg^2+^ transporters from two distinct families, the CorA-like MGT10 and the CorC-like MGR8 and MGR9. Both families play an essential role for the plant since the single knockout of MGR10 and the double knockout mutation MGR8 and MGR9 result in impaired chloroplast development. Nevertheless, how Mg^2+^ homeostasis is regulated by MGR8, MGR9, and MGT10 in the plant chloroplast and if their function is conserved in algae remained unknown. Our study provides several lines of evidence for a differential role of the three transporters in the chloroplast function. In addition, we identified an MGT10 homolog in Chlamydomonas, MRS4, that is essential for photosynthesis and cell growth.

Firstly, we demonstrate distinct expression patterns of the three transporters in Arabidopsis plants grown under different external Mg^2+^ concentrations. Their expression pattern in various organs was previously analyzed in WT plants grown at standard Mg^2+^ supply (Drummond et al., 2006; Sun et al., 2017; Zhang et al., 2022), but neither at low or high concentrations nor in mutants. In plants grown at standard Mg^2+^ concentration, *MGR8* and *MGT10* genes were highest expressed in leaves (**Figure 3A**), indicating a function in chloroplast Mg^2+^ transport. *MGR9* was upregulated in Arabidopsis plants grown in conditions of Mg^2+^ deficiency (**Figures 3B, C**). Heterologous expression of MGR9 rescued the growth of *E. coli* TM2 cells at a lower Mg^2+^ concentration than MGR8 (**Figures 2A, B**). Therefore, we propose that MGR9 is required for chloroplast function in low Mg^2+^ environments.

The Mg^2+^ concentration in the chloroplast stroma is important for regulation of RuBisCO enzyme activity (Mott and Berry, 1986). In our study, we observed a 38-21% reduced Mg^2+^ content in the chloroplast of *mgr8, mgr9*, and *mgt10*, suggesting a role for all three transporters in magnesium uptake into the chloroplast. However, this suggestion does not explain why the Mg^2+^ content of *mgt10mgr8-2* was 17% higher than that of WT (**Figure 6**). Therefore, further studies are required to elucidate the directionality of transport for the three proteins in Arabidopsis. The CO_2_ fixation activity and plant biomass were non-significantly different among genotypes (**Supplementary Figures S9**, **S12**). We postulate that the observed alterations in the Mg^2+^ content in the mutant chloroplasts were mild, and therefore did not impact the carboxylase activity of RuBisCO. All studied mutants displayed a lower NPQ due to a lower ΔpH at increasing light intensities when grown at low and standard Mg^2+^ concentrations (**Figures 5A, B, D, E**). These data indicate that MGR8, MGR9, and MGT10 are involved in building up the pH gradient to rapidly activate NPQ without largely affecting the electron transport through photosystems and overall PMF size. The observation of a lower ΔpH in all analyzed mutants lacks an obvious explanation, as MGTs and MGRs are known to facilitate the direct transport of Mg^2+^ ions and not H^+^. From previous studies, we know that a Mg^2+^ influx can lower the pH of the stroma due to the activation of a reversible (Na^+^)K^+^/H^+^ exchange across the envelope (Huber and Maury, 1980; Wu et al., 1991; Wu and Berkowitz, 1992). Our data show altered Na^+^ and K^+^ homeostasis in the chloroplasts of the *mgt10mgr8-2* double mutant (**Figures 6B, C**), which also displayed the most different NPQ and ΔpH from WT in conditions of excess light (**Figures 5A, B, D, E**). Thus, potentially the studied envelope Mg^2+^ transporters affected the PMF partitioning to ΔpH indirectly *via* cation/H^+^ exchangers. Such a possibility is supported by reduced NPQ and ΔpH in loss of function Arabidopsis mutants for the envelope K^+^/H^+^ exchangers KEA1 and KEA2 in conditions of excess light (Kunz et al., 2014). Future studies with double mutants of an Mg^2+^ transporter and cation/H^+^ exchanger may provide more insights into the potential coordination of ion transport across the chloroplast envelope impacting pH homeostasis.

An alternative explanation for the altered mineral content could be related to the phylogenetic grouping of MGR8, MGR9 and MGT10. Our protein sequence alignment indicates that MGR8 and MGR9 are not CNNM- but CorC-like transporters, whereas MGT10 shares the closest evolutionary history with CorA (**Figure 1**, **Supplementary Figure S1A**). MGR8 transports Mg^2+^ by a distinct mechanism as compared to CNNMs, as evidenced by the heterologous expression of several mutant lines (**Figures 2E, F**). Furthermore, we demonstrate that in contrast to MGT10, the Mg^2+^ transport activity of MGR8 and MGR9 was not inhibited by Al^3+^ (**Figures 2C, D**), implying a distinct transport mechanism than for CorA-like members. Structurally, CorC is a Na^+^-dependent transporter, whereas CorA adopts a channel-like architecture with an ion-conducting pore (Franken et al., 2022; Jin et al., 2022). The Mg^2+^ transport activity of CorC is driven by the Na^+^ gradient, and an Asparagine residue was identified to play an essential role (Huang et al., 2021). Both MGR8 and MGR9 contain this residue (**Supplementary Figure S1B**), indicating possible coordination of transport between Mg^2+^ and Na^+^ across the chloroplast inner envelope. Based on our protein sequence alignment, we postulate that MGT10 might function as a Mg^2+^ ion channel, whereas MGR8 and MGR9 function as Na^+^-dependent Mg^2+^ transporters. Future structural and functional analyses are required to fully understand the transport mechanism of all three transporters.

Chloroplasts are organelles with a high demand for Mg^2+^ to ensure the maintenance of photosynthetic activity. So far, no Mg^2+^ transporter is known in the chloroplast of unicellular or multicellular algae. Our study found one MGT10-like homolog (MRS4) in *Chlamydomonas reinhardtii* and *Volvox carteri* (**Supplementary Figure S3**) but no chloroplast MGR8 or MGR9-like sequences. Our findings from the analyses of CrMRS4 provide strong support for a Mg^2+^ transport function and critical role in photosynthesis and cell growth. We found a low NPQ induction and thus reduced capacity for photoprotection in the *mrs4* mutant (**Figure 7E**). We propose that this enhanced sensitivity to light is a result of a higher proportion of closed PSII centers and the potential malfunctioning of the PSII complex (**Figures 7B–D**). The partial rescue of the *mrs4* phenotype (**Figure 7**) by complementation with the *VcMRS4* implies that the functionality of the two MRS4 proteins may be partly different. Even supplementation with excess Mg^2+^ during growth did not fully rescue the *mrs4* phenotype, potentially due to the presence of excess Mg^2+^ inside the chloroplast (**Supplementary Figure S16**).

Our TEM results show that thylakoid stacking was affected in *mgt10* but not in *mgr8* or *mgr9* (**Figure 4**). Interestingly, the observation of an opposite pattern in grana size, i.e., smaller in *mgr8mgr9* (Zhang et al., 2022) and larger in the peculiar plastids in *mgt10* (**Figures 4D, F**; Sun et al., 2017), implies different roles of the two families of transporters. Knock-out of the MGT10 homolog *MRS4* in Chlamydomonas resulted in more stacked thylakoids that contained more layers (**Supplementary Figure S15**). This morphotype is very similar to the macro-grana observed in the Arabidopsis *mgt10* mutant, indicating an evolutionarily conserved function for members of this protein family. There are several other common features between the *mgt10* and *mrs4* mutants, including the reduced F_v_/F_m_ and NPQ (**Figures 4A, B**, **7C, E** and **Supplementary Table S1**). These perturbations in photosynthetic efficiency can be attributed to the macro-grana thylakoid ultrastructure, which likely disturbs the organization of LHCII-PSII complexes and movement of damaged complexes during repair in light conditions (Sun et al., 2017). Taken together, our results demonstrate that the three Arabidopsis magnesium transporters have differential roles in maintaining chloroplast magnesium homeostasis. Future research should focus on investigating the mechanisms underlying the functional coordination among these three magnesium transporters, as well as their interactions with other chloroplast transporters, to provide a more comprehensive understanding of magnesium homeostasis in plant cells.

## 4 Materials and methods

### 4.1 Plant material and growth conditions

*Arabidopsis thaliana* wild type (Columbia-0) and the T-DNA insertion lines *mgr8-1* (SALK_074964), *mgr8-2* (SALK_007335), *mgr9-1* (SALK_061515), and *mgr9-2* (SALK_087652) were obtained from the SALK collection (Alonso et al., 2003), and have been described by Zhang et al. (2022). The *mgt10* (GABI_764F12) was obtained from the GABI-KAT collection (Rosso et al., 2003) and is a heterozygous knockdown mutant. Genotyping of the T-DNA insertion lines was done by PCR/RT-PCR with gene-specific primers (**Supplementary Table S2**). The double *mgt10mgr8-2* mutant was obtained by crossing *mgt10* and *mgr8-2*. Wild type (WT) plants and mutants were grown hydroponically for 6-7 weeks in a growth chamber (CLF PlantMaster; Plant Climatics, Wertingen, Germany) using a daily cycle of 16 h of light (120 µmol photons m^−2^ s^−1^) at 21°C and 8 h of dark at 19°C at a relative humidity of 70%. The nutrient solution was prepared as described (Gibeaut et al., 1997; Conn et al., 2013) with the modification that MgSO_4_ was used at the concentration of 0 (low Mg^2+^), 0.75 (standard Mg^2+^) and 3 mM (high Mg^2+^).

### 4.2 *Chlamydomonas reinhardtii* material and cultivation conditions

Wild type *Chlamydomonas reinhardtii* (strain CC-4533) and the *mrs4* mutant (strain LMJ.RY0402.244553) were obtained from the CLiP library of the Chlamydomonas Resource Centre (https://www.chlamylibrary.org/). The strains were maintained in darkness on agar plates (1.2% w/v) prepared with Tris-Acetate-Phosphate (TAP) containing 0.4 mM MgSO_4_. For genotyping, DNA was extracted, and PCR was performed with the appropriate combination of primers (**Supplementary Table S2**). For the spot tests, a loopful of culture was transferred to TAP medium and grown in darkness for 2 days. The cells were then resuspended to a density of 5 x 10^5^ cells mL^-1^ in TAP which was enriched or not with 2 or 5 mM MgSO_4_, spotted at different dilutions on TAP agar plates containing corresponding MgSO_4_ concentration, and allowed to grow in darkness at 20°C. From the same inoculum, cells were resuspended in minimal Tris-Phosphate (TP) medium which was enriched or not with 2 or 5 mM MgSO_4_, spotted on TP plates, and allowed to grow at 20°C using a daily cycle of 16 h of light (100 µmol photons m^−2^ s^−1^) and 8 h of dark. For photosynthetic analyses, strains were grown in liquid TP medium at an initial density of 1x 10^6^ cells mL^-1^ in a volume of 50 ml for 4 days using a daily cycle of 16 h of light (100 µmol photons m^−2^ s^−1^) and 8 h of dark.

### 4.3 Phylogenetic analysis

Whole genome sequence data from a representative set of plant species were downloaded from Phytozome v13 (https://phytozome-next.jgi.doe.gov/). Bacterial data were downloaded from NCBI (https://www.ncbi.nlm.nih.gov/) and sequences of the diatom *Thalassiosira pseudonana* from the JGI Genome portal (https://genome.jgi.doe.gov/portal/). The amino acid sequences were then queried using BLASTp v2.2.28+ (Camacho et al., 2009) and MAFFTv6.843b (Katoh and Toh, 2008) to identify homologs of the Arabidopsis MGR9 (At1g55930) and MGR8 (At3g13070) (https://github.com/topel-research-group/misc/blob/master/blast_and_align.py). Phylogenetic analyses of the identified homologs were then performed using MrBayes v3.2.6 (Ronquist et al., 2012) for 1 million generations (Brassicacese analysis) and 2 million generations (Plants, bacteria, diatom, green algae and human sequences), respectively, after which the Average Standard Deviation (AvgStdDev) was significantly low, and the analyses were assumed to have converged. The first 25% of the tree samples were discarded and the remaining trees were summarized in majority consensus trees (see **Figure 1**, **Supplementary Figure S2**).

The following protein sequences were used for constructing the phylogenetic tree in **Supplementary Figure S1A**: AtMGT10 (NP_568424.1), EcCorA (P0ABI4), EcCorC (P0AE78), TpCorC (WP_060384576.1), AtMGR8 (NP_187914.1), AtMGR9 (NP_187914.1), hCNNM2 (Q9H8M5) and hCNNM4 (Q6P4Q7). For the construction of the phylogenetic tree of CrMRS4 (Cre50g761497), amino acid sequences for Mg^2+^ transporters were downloaded from the plant membrane protein database (http://aramemnon.uni-koeln.de/) for *Arabidopsis thaliana*, and from Phytozome v13 (https://phytozome-next.jgi.doe.gov/) for *Chlamydomonas reinhardtii* (v6.1) and *Volvox carteri* (v2.1). The sequences were aligned using ClustalW in MEGA11 (Tamura et al., 2021) and the phylogenetic trees were generated using the neighbor-joining method with 500 bootstraps and the default settings of MEGA11.

### 4.4 Complementation of *E. coli* TM2 mutant with Arabidopsis *MGR8* and *MGR9*

The Arabidopsis sequences that encode for MGR8 (stock no. GPSO-0186) and MGR9 (BRC no. pda13388) were obtained from the Arabidopsis Biological Resource Center (http://abrc.osu.edu/) and the RIKEN BRC through the National BioResource Project of the MEXT/AMED, Japan, respectively. The MGR8 and MGR9 proteins contain N-terminal extensions. The ChloroP1.1 algorithms predicted a chloroplast leader and transit peptide cleavage sites at amino acid 71 of MGR8 and amino acid 72 of MGR9, respectively (**Supplementary Figure S1B**). The cDNA fragments that encode MGR8 from F72 to the C-terminal Q661 residue and MGR9 from L73 to the C-terminal E653 residue were subcloned in frame at the *Nco*I site of the pTV118N vector (TakaraBio, Japan) for the *E. coli* complementation assay (Ishijima et al., 2015). To generate various mutants, inverse PCR-based mutagenesis was performed using appropriate primer sets (**Supplementary Table S2**), and the obtained mutant cDNAs were subcloned into the pTV118N vector. All plasmid sequences were confirmed by DNA sequencing.

The plasmids containing the *MGR8* and *MGR9* wild type and mutant cDNAs were transformed into *E. coli* mutant TM2 (Δ*corA ΔmgtA ΔyhiD*) cells (Ishijima et al., 2015). TM2 transformed with an empty pTV118N vector was used as a negative control. Culture growth was monitored at 37°C with an ODBOX-c OD-Monitor (TAITEC, Japan).

### 4.5 Quantitative real-time PCR analysis

Total RNA was isolated from two-week-old seedlings and plant tissues of 6-week-old plants with an E.Z.N.A. R6827-01 Plant RNA kit (Omega Bio-Tek, GA, USA) and residual DNA was removed with E1091 DNAse (Omega Bio-Tek). cDNA was synthesized using 500 ng of total RNA through iScript cDNA Synthesis Kit (Bio-Rad, Hercules, CA, USA). Quantitative real-time PCR analyses were conducted with a SsoAdvanced Universal SYBR Green Supermix on a CFX96 Touch Thermal Cycler (Bio-Rad). Fifty ng of cDNA was used as qPCR template in 10 µl reactions. Amplifications were done in a two-step PCR with the following conditions: initial denaturation for 2 min at 95°C, followed by 40 cycles of denaturation for 5 s at 95°C, annealing for 30 s at 60°C, and extension for 10 s at 72°C. After amplification, melt-curve analyses were performed for all primers. Gene-specific primers used were ordered from Bio-Rad (**Supplementary Table S3**). ΔCq method (2^-ΔCq^) was used to calculate relative expression using *PEX4* and *ACTIN8* as the reference genes.

### 4.6 Immunoblotting

Total protein extracts were prepared from 100 mg snap frozen leaves ground in 500 μL PEB buffer (AS08300, Agrisera, Umeå, Sweden) and centrifuged to remove insolubilized material. Intact chloroplasts were prepared from 50 g fresh leaves ground in 50 mM HEPES-KOH (pH 7.8), 300 mM sorbitol, 2 mM Na_2_EDTA, and 5 mM ascorbic acid, centrifuged and loaded on Percoll 40/75% gradients. Intact chloroplasts were collected following centrifugation of the gradients at the 40%-75% interface, washed, and resuspended in 50 mM HEPES-KOH (pH 7.8), 300 mM sorbitol, and 2 mM Na_2_EDTA. Intactness was verified by microscopy and the number of chloroplasts was counted using a hemocytometer. Thylakoid and envelope membranes were prepared from lysed chloroplasts, and centrifuged at 3000 *g* and 150,000 *g*, respectively. Protein concentration was determined using a Bradford assay (Bio-Rad). Equal amounts of protein (8 μg) were separated on 4-15% (w/v) SDS-PAGE gels (Bio-Rad) and transferred to polyvinylidene difluoride (PVDF) membranes. Nonspecific bindings on the membranes were blocked with 5% (w/v) milk. An antibody was generated by Agrisera in rabbit against the EHVLADNSKKQQ C-terminal peptide of MGR8 (**Supplementary Figure S1B**). The rabbit anti-Tic40 and Lhcb1 antibodies were purchased from Agrisera (AS10709, AS01004). Following incubation with a goat anti-rabbit secondary antibody (Bio-Rad), the chemiluminescent signal on the blots was detected using Clarity and Clarity Max ECL substrates (Bio-Rad).

### 4.7 Transmission electron microscopy

The fixation and embedding of 1 x 2 mm Arabidopsis leaf pieces for TEM were carried out as described (Böszörményi et al., 2020). In the case of WT and *mgt10*, TEM samples close to leaf veins were also fixed. Fixation and embedding of Chlamydomonas cells were done following the protocol of Skepper (2000), with gentle centrifugation following each step to collect the cells. Briefly, the Chlamydomonas cells of the liquid media were centrifuged at 1000 *g* for 5 min at room temperature, washed with 10 mL fresh TAP/TP medium, then centrifuged again as described above. The pellet was then resuspended in 1 mL of 2.5% glutaraldehyde in 50 mM HEPES (pH 7.4) and fixed for 1 h at room temperature on a tube rotator. Cells were then centrifuged at 5000 *g* for 3 min at room temperature. The pellet was washed for 3 x 5 min with ddH_2_O, with centrifugation with the above parameters after each washing step. Samples were postfixed for 1-2 hrs at 4°C in 1 mL 1% OsO_4_, 1.5% (w/v) K_3_[Fe(CN)_6_], and 2 mM CaCl_2_. After centrifugation as above, samples were washed 4 x 5 min with ddH_2_O. Samples were then stained for 1-2 h at room temperature in 1 mL Bulk Stain solution (2% uranyl acetate in 0.05 M maleate buffer, pH 5.5). After staining, samples were again centrifuged as above and then washed 3 x 5min with ddH_2_O. After dehydration in graded ethanol series, samples were transferred to acetonitrile and then embedded in epoxy resin (Quetol 651, nonenyl succinic anhydride - NSA, methyl-5-norbornene-2,3-dicarboxylic anhydride - NMA, and catalyst dimethylbenzylamine - BDMA). The resin-embedded Chlamydomonas pellet samples were polymerized in Eppendorf tubes that were then cut into smaller pieces which were then re-embedded in Durcupan ACM epoxy resin (Fluka Chemie AG) in blocks suitable for ultrathin sectioning. A Reichert Jung ULTRACUT E microtome was used for ultrathin (70 nm) sectioning of all resin-embedded samples. After a 5-min staining with 5% uranyl acetate dissolved in methanol, and a subsequent 5-min-long treatment with Reynold’s lead citrate solution, the copper grids holding the sections were investigated using a JEOL JEM 1011 (JEOL Ltd., Japan) at 80 kV accelerating voltage as described (Dukic et al., 2019). An Olympus Morada CCD camera (Olympus Optical Co. Ltd., Japan) was used to take digital images. At least 25 randomly chosen cell sections were studied for each sample and representative images for all samples were chosen for the preparation of **Figure 4** and **Supplementary Figure S15**.

### 4.8 Kinetics of chlorophyll *a* fluorescence induction

Fast kinetics of Chl *a* induction in Arabidopsis leaves were recorded on 30 min dark-adapted plants by applying saturating red actinic light of 3,600 µmol photons m^−2^ s^−1^ for 1 s using a Handy-PEA (Hansatech, UK) fluorometer. Initial F_0_ and F_m_ fluorescence values were determined by the saturating pulse. The maximal quantum yield of PSII photochemistry (F_v_/F_m_) was calculated as (F_m_-F_0_)/F_m_ using Hansatech PEA Plus v1.10 software according to Strasser et al. (2004). The slow kinetics of Chl fluorescence induction and P700 oxidation-reduction of Arabidopsis were simultaneously recorded with a Dual-PAM-100 equipped with DUAL-DB and DUAL-E emitter-detector module (Walz, Effeltrich, Germany). The kinetics were recorded on attached leaves of 30 min dark-adapted plants exposed to fluctuating red actinic light as follows: 10 min in low light (70 µmol photons m^−2^ s ^−1^) followed by 3 min in high light (650 µmol photons m^−2^ s^−1^) and then again 3 min in low light. Saturating red pulses of 5,000 µmol photons m^−2^ s^−1^ and 800 ms duration were applied for determination of the maximal fluorescence yield in the dark state (F_m_) and during the period with actinic light (F_m_′). NPQ and Y(II) were calculated based on changes in Chl fluorescence as (F_m_–F_m_^′^)/F_m_^′^ and (F_m_^′^–F)/F_m_^′^, respectively, according to Genty et al. (1989). Y(I) was calculated from absorbance changes at 830 nm according to Klughammer and Schreiber (1994).

To measure PSII activity in Chlamydomonas, cells grown in TP in the light were resuspended to 30 µg Chl mL^-1^ and incubated with agitation at 50 rpm in darkness for 15 min. Kinetics of Chl fluorescence induction were recorded using DUAL-PAM-100 during actinic illumination at 2,500 μmol m^-2^ s^-1^ for 8 min followed by 4 min of dark relaxation. Saturating red pulses of 3,000 µmol photons m^−2^ s^−1^ were applied to the samples in a cuvette under continuous stirring for determination of F_m_ and F_m_^′^. The maximal photochemical efficiency of PSII (F_v_/F_m_), NPQ and Y(II) were calculated as above. Rapid light response curves of electron transport rate were recorded using photosynthetically active radiation increasing stepwise from 26 to 2,015 μmol photons m^-2^ s^-1^.

For imaging analysis of Chl fluorescence using FluorCam 800 MF (Photon System Instruments, Drasow, Czech Republic), 1 mL of Chlamydomonas cells containing 30 µg Chl were dark-adapted, transferred onto a 12-well cell culture plate (Nunc, Thermo Fisher Scientific, USA) and analyzed using default quenching 1 protocol with actinic white light at 100 µmol photons m^−2^ s^−1^ exposure for 6 min followed by 2 min in darkness. Saturating flashes of white light at 3,000 μmol photons m^−2^ s^−1^ were applied after every min of light/dark with a shutter speed of 10 μs and sensitivity of 50%.

### 4.9 Electrochromic shift

ECS measurements were carried out using the Dual-PAM-100 system equipped with a P515/535 module. Arabidopsis plants were first 30 min dark-adapted and then exposed to fluctuating red light as described in section 4.8. ECS was measured at the end of each low-to-high light and high-to-low light transition. PMF size was calculated as the difference between the ECS signal in light and the minimum value of the ECS signal after the light was turned off. Calculation of PMF partitioning to ΔpH and ΔΨ was performed using the steady-state time point of the ECS signal in darkness (Cruz et al., 2001). Before each ECS measurement, three saturating 50-μs actinic red flashes of 200,000 μmol photons m^−2^ s^−1^ were applied to determine the ECS_ST_; subsequently, the ECS_ST_ amplitude was used to normalize the ECS signal before the calculation of PMF size and partitioning values.

For ECS in Chlamydomonas, cells grown in TAP in darkness were resuspended at 30 µg Chl mL^-1^ and incubated with agitation at 50 rpm in darkness for 15 min before being layered on a glass slide and exposed to actinic red light for 2 and 7 min. The light was switched off and decay kinetics were measured to calculate ECSt, ECSst, PMF size and partitioning to ΔpH and ΔΨ as for Arabidopsis.

### 4.10 Mineral content measurement by inductively coupled plasma mass/optical emission spectrometry

Plant shoot samples were collected directly from the hydroponic system after 6-7 weeks, washed twice with double distilled water (ddH_2_O), and then dried in the oven for 3 days at 80°C. The dried sample was crushed to a fine powder using a ceramic mortar and pestle. A 100 mg subsample was digested with 70% (v/v) HNO_3_ and 30% (v/v) H_2_O_2_ in a ratio of 3:1 (Chen A. et al., 2020) at 240°C and 200 bars for 15 min in a pressurized microwave oven (Milestones SRL, Italy). After dilution to 3.5% acid, the element concentrations were determined using ICP-OES (5100; Agilent Technologies) and data were processed using Agilent ICP Expert software (Hansen et al., 2009). Similarly, a subsample of chloroplasts purified on a 40%/75% Percoll gradient and adjusted to contain 10^9^ chloroplasts mL^-1^ were digested and analyzed using ICP-MS (7900; Agilent Technologies) and data were processed using Agilent Masshunter software, as the low element concentrations required a more sensitive detector than the one used for plant tissues.

To determine the mineral content in Chlamydomonas, cell colonies maintained on 1.2% (w/v) agar TAP plates were used to inoculate liquid TAP medium for growth in darkness and shaking for three days. Early log phase cultures were harvested and washed with either TAP or TP medium followed by resuspension in the corresponding medium to reach 0.5 x10^5^ cells mL^-1^. The cells were grown either in TAP in darkness or TP under continuous illumination (100 μmol photons m^-2^ s^-1^) and shaking for four days. Samples (in triplicate) containing 2 x10^7^ cells (~800 mg) were harvested and washed twice in 5 mM HEPES (pH 7.0) and 2 mM EDTA before collection and air-drying of the cell pellet. Pelletized cells were digested in screw-capped Teflon vials on a hot plate at elevated temperatures (25-75°C) using a 5:2 mixture of HNO_3_ (68-70% v/v) and H_2_O_2_ (30% v/v)). Samples were then dissolved in 2% HNO_3_ and analyzed for cation ratios (Mg^2+^/Na^+^, K^+^/Na^+^, and Ca^2+^/Na^+^) on an ICP-MS (ICAP-Q; ThermoScientific). Elemental ratios were quantified using a series of externally calibrated standards (Inorganic Ventures Co.) and analytical uncertainties were estimated at ~5-10% (1σ) based on repeat measurements of the calibration standards.

### 4.11 CO_2_ fixation

Net photosynthesis rates in terms of CO_2_ fixation were determined using Li-COR portable Photosynthesis System Li-6400 (Lincoln, Nebraska, USA). Plants were first light-adapted for at least 1 h in the growth chamber and then exposed in the gas exchange chamber to broad spectrum light generated with red, green, and blue LEDs at 120 and 700 µmol photons m^−2^ s^−1^ in atmospheric CO_2_ (440 µmol mol^−1^) for approximately 5 min or until a steady state was reached. Data were normalized to leaf area determined using ImageJ.

### 4.12 Complementation experiments of Chlamydomonas *mrs4* mutant with Volvox *MRS4*

The Chlamydomonas *MRS4* gene was incomplete in the v5.5 genome (Cre50.g761497), and trials to obtain it by TAIL-PCR have failed. For complementation experiments of *mrs4*, we have chosen to clone its homolog in *Volvox carteri* (Vocar.0040s0086) using the pipeline described by Mackinder et al. (2017). Briefly, the open reading frame was amplified from Volvox genomic DNA by PCR from two fragments (2-3 kbp each) using Phusion High-Fidelity DNA polymerase (New England BioLabs) and primers given in **Supplementary Table S2**. The PCR products were purified from agarose gels, re-assembled, and cloned in-frame with a C-terminal Venus-3×FLAG in pLM005 by Gibson assembly (New England BioLabs). The construct was linearized by EcoRV-HF and verified by sequencing. For transformation of the *mrs4* mutant, cells were cultured in TAP liquid medium at 23°C under light at 150 µmol photons m^−2^ s^−1^ to 1-2 x 10^6^ cells mL^-1^. The cells were washed and suspended at 2 x 10^8^ cells mL^-1^ in MAX Efficiency Transformation reagent (Invitrogen) together with the linearized plasmid and by electroporation using a NEPA21 electroporator (NEPA GENE). The transformants were plated on TP agar supplemented with 25 μg mL^-1^ and grown under light (150 μmol photons m^−2^ s^−1^) for 7 days. Those transformants showing better growth than the *mrs4* mutant were screened by PCR with primers given in **Supplementary Table S2** to verify the correct insertion of *VcMRS4*.

## Data availability statement

The original contributions presented in the study are included in the article/**Supplementary Material**. Further inquiries can be directed to the corresponding author.

## Author contributions

ED, SI, and CS conceived the study and designed the experiments. ED carried out the ECS, Chl fluorescence, CO_2_ fixation measurements, and qRT-PCR. KAvM carried out the localization western blots. KMS and CS performed the Chlamydomonas work. KvM, KMS, and MT performed the phylogenetic analyses. KF, SS, and SI run the heterologous characterization in *E. coli*. ED and CS fixed and embedded the samples for TEM. KS performed the TEM analyses. JHe generated the *mgt10mgr8-2* double mutant. TH and SH performed mineral analyses of Arabidopsis samples. JHi performed the mineral analyses of Chlamydomonas cells. ED, KvM, and KMS performed the statistical analyses. KvM, SI, ED, KMS, and CS wrote the manuscript. All authors helped to edit the manuscript and approved the submitted version.
