# Supplementary material for: Chloroplast magnesium transporters play essential but differential roles in maintaining magnesium homeostasis
Source: Front Plant Sci. 2023 Aug 23;14:1221436. doi: 10.3389/fpls.2023.1221436 (PMC10484576; doi:10.3389/fpls.2023.1221436)
Supplement: Supplementary file 1 [file DataSheet_1.pdf]

Supplementary Material

Chloroplast magnesium transporters play essential but differential roles in maintaining magnesium homeostasis

Emilija Dukic[1†], Kim A. van Maldegem[1†], Kashif Mohd Shaikh[1†], Kento Fukuda[2], Mats Töpel[3,4], Katalin Solymosi[5], Jonna Hellsten[1], Thomas Hesselhøj Hansen[6], Søren Husted[6], John Higgins[7], Satoshi Sano[2], Sumio Ishijima[2], and Cornelia Spetea[1*]

[1]Department of Biological and Environmental Sciences, University of Gothenburg, 40530 Gothenburg, Sweden.

[2]Graduate School of Life and Environmental Sciences, Kyoto Prefectural University, 606-8522 Kyoto, Japan

[3]Department of Marine Sciences, University of Gothenburg, 40530 Gothenburg, Sweden

[4]IVL Swedish Environmental Research Institute, 41133 Gothenburg, Sweden

[5]Department of Plant Anatomy, ELTE Eötvös Loránd University, 1117 Budapest, Hungary

[6]Copenhagen Plant Science Centre, Department of Plant and Environmental Sciences, University of Copenhagen, 1871 Frederiksberg C, Denmark

[7]Department of Geosciences, Princeton University, 8544 Princeton, NJ, USA.

† These authors contributed equally to this work and share first authorship

*** Correspondence:**
Corresponding Author
cornelia.spetea.wiklund@bioenv.gu.se

Supplementary Figures

A

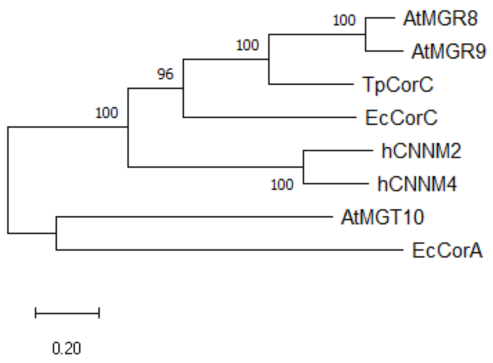

B

```
AtMGT10    ------------------------------------------------------------    0
EcCorA     ------------------------------------------------------------    0
EcCorC     ------------------------------------------------------------    0
TpCorC     ------------------------------------------------------------    0
AtMGR8     -------------------------MMGMALELSVLGR----------------------    13
AtMGR9     ----------------------------MELDLSVLGR----------------------    10
hCNNM2     MIGCGACEPKVKMAGGQAAAALPTWKMAARRSLSARGRGILQAAAGRLLPLLLLSCCCGA    60
hCNNM4     ---------MAPVGGGG-------------RPVGGPARGRLLLAAPVLLV---LLWAL--    33

AtMGT10    -----------------------------------------MALTP----IPSTFTSLFN    15
EcCorA     ------------------------------------------------------------    0
EcCorC     ------------------------------------------------------------    0
TpCorC     ------------------------------------------------------------    0
AtMGR8     -----------SVIDSKTLN-L-KRYGQKSKLSGRFLPRAELH-CPVALSSSKHSNLSFR    59
AtMGR9     -----------SFIVTRRNSSITRPCIQSSNFSVRVLQ-RNKH-RPLCFSTNPSNSSFIR    57
hCNNM2     GGCAAVGENEETVIIGLRLEDTNDV-SFMEGGALRVSERTRVKLRVYGQNINNETWSRIA    119
hCNNM4     -GARGQGSPQQGTIVGMRLASCNKSCGTNPDGIIFVSEGSTVNLRLYGYSLGNISSNLIS    92

AtMGT10    FSDHSPYPSPSLHYLLPGSSPSFSLQL----------SALSRTPIYF-------------    52
EcCorA     ------------------------------------------------------------    0
EcCorC     ------------------------------------------------------------    0
TpCorC     ------------------------------------------------------------    0
AtMGR8     FRRSCEFSYRSRFMLFSSSQCHEGSQR-KSDSGEK---ELESIKVLL-----KRGIVIGA    110
AtMGR9     FQKGCDFSHRCQFVVLSATGDHVGISQ-KHSDSTE---KVDSIRILL-----KRGIVLGA    108
hCNNM2     FTEHERRRH------SPGERGLGGPAPPEPDSGPQRCGIRTSDIIILPHII-LNRRTSGI    172
hCNNM4     FTEVDDAET------LH---------------KSTSCLELTKDLVVQQLVNVSRGNTSGV    131

AtMGT10    --EALKVLSRSKCFAKSPTTAEDFVGDYESLNVSDDDDDGSDSNSSDGDNGGRDDSKK--    108
EcCorA     ------------------------------------------------------------    0
EcCorC     ------------------------------------------------------------    0
TpCorC     ------------------------------------------------------------    0
AtMGR8     L----------VCG----------VFLYGCQKVLA-------------------SAGV--    129
AtMGR9     V----------VCG----------VLFYGCGKVLA-------------------STSV--    127
hCNNM2     IEIEIKPLRKMEKS----------KSYYLCTSLSTPALGAGGSGSTGGAVGGKGGSGVAG    222
hCNNM4     LVVLTKFLRRSESM----------KLYALCTRAQ--------------------------    155

AtMGT10    -IDSSSS------S-SSSDS-----TSLGIREPVYEVV-----EVKATGAISTRKINRRQ    150
EcCorA     ------------------------------------------------------------    0
EcCorC     ------------------------------------------------------------    0
TpCorC     -MDRP---PSRWLLLLALGTPALAQGEAASPENPWLWA-VLVLLLALSAFFSASE-----    50
AtMGR8     -VEAGYEVFGQSVVLFKNALPKIYQVLTVLREQ---GL-ILAALLSLSAFFSMAE-----    179
AtMGR9     -VDVA---FSKSILLLKNAWPKTSQVLKVLREQ---GL-ILAVLLGLSAFFSMAE-----    174
hCNNM2     LPPPPWA-ETTWIYHDGEDTKMIVGEEKKFLLPFWLQVIFISLLLCLSGMFSGLN-----    276
hCNNM4     -PDG------PWLKWTDKDSLLFMVEEPGRFLPLWLHILLITVLLVLSGIFSGLN-----    203

AtMGT10    LLKSSGLRPRDIRSVDPSLFMTNSVPSLLVREHAILLNLGSLRAIAMRDRVLIFDYNRRG    210
EcCorA     ------------------------------------------------------------    0
EcCorC     ------------------------------------------------------------    0
TpCorC     -TAITTLYPWKLKELA---------------------E--SKNG--------PFRLLAED    78
AtMGR8     -TSITTLWPWKVRELA---------------------EKEPENG--------VFRMLRSD    209
AtMGR9     -TSITTLWPWKVRELA---------------------EKEPENG--------VFRMLRSD    204
hCNNM2     -LGLMALDPMELRIVQ---------------------NCGTEKE---KNYAKRIEPVRRQ    311
hCNNM4     -LGLMALDPMELRIVQ---------------------NCGTEKE---RRYARKIEPIRRK    238

AtMGT10    GRAFVDTLM-----------------------------------------PRLNPRSMNG    229
EcCorA     ------------------------------------------------------------    0
EcCorC     ---------------------------------------------------------MSD    3
TpCorC     ITRFLTTILVGNNLVNIAATALVTELATQAFGSAGVGVATGAMTFLILFFGEITPKSLAV    138
AtMGR8     VTRFLTTILIGTTVVNIAATALVTEAATAIFGEAGVSAATGLMTVAILLLTEITPKSVAV    269
AtMGR9     VTRFLTTILIGTTVVNIAATALVTKAATAIFGEAGVSAATGVMTVAILLLTEITPKSVAV    264
hCNNM2     GNYLLCSLLLGNVLVNTTLTILLDDIAGS----GL--VAVVVSTIGIVIFGEIVPQAICS    365
hCNNM4     GNYLLCSLLLGNVLVNTSLTILLDNLIGS----GL--MAVASSTIGIVIFGEILPQALCS    292
```

```
AtMGT10      GPS---------------------------------------------------------   232
EcCorA       ------------------------------------------------------------   0
EcCorC       DNSHSSDT------------IS----NKKGFFSLL-------LSQLFHGEPK-NRDELLA   39
TpCorC       HHAEAIARLAAWPIYGLSVLFY----PVGRFFSLVSGGLLRLLGLEPRGTPLVSEEELKL   194
AtMGR8       HNAQEVARIVVRPVAWLSLVLY----PVGRIVTYLSMGILKILGLKGRSEPYVTEDELKL   325
AtMGR9       HNAQEVARIVVRPVAWLSLILY----PVGRVVTYLSMGILKILGLKGRSEPYVTEDELKL   320
hCNNM2       HGLAVGANTIFLTKFFMMMTFPASYPVSKLLDCV-------LGQEIG--TVYNREKLLER   416
hCNNM4       RHGLAVGANTILLTKFFMLLTFPLSFPISKLLDFF-------LGQEIR--TVYNREKLME   343

AtMGT10      -----------MPFELEAVESALISRIQRLEQRLMD-IEPRVQAL---------------   265
EcCorA       MLS------------------AFQLENNRLTRLEVEESQPLVNAVWIDLVEPDDDERLRV   42
EcCorC       LIRDSGQNDLIDEDTRDMLEGVMDIADQRVR----DIMIPRSQMITLKRNQTLD-E---C   91
TpCorC       ILAGAEESGAIQPQEEEMIHSILELEETPVR----EIMTPRVEMVAIEDEATLE-D---L   246
AtMGR8       MLRGAELSGAIEEEEQDMIENVLEIKDTHVR----EVMTPLVDVVAIDASASLV-D---F   377
AtMGR9       MLRGAELSGAIEEEEQDMIENVLEIKDTHVR----EVMTPLVDVVAIDGSGSLV-D---F   372
hCNNM2       MLRVTDPYNDLVKEELNIIQGALELRTKTVE----DVMTPLRDCFMITGEAILDFN---T   469
hCNNM4       MLKVTEPYNDLVKEELNMIQGALELRTKTVE----DIMTQLQDCFMIRSDAILDFN---T   396
:      :     :      : .

AtMGT10      ------------------------------------------------------------   265
EcCorA       QSELGQSLATRPELEDIEASARFFEDDDGLHIHSFFF------FEDAEDHAGNSTVAFTI   96
EcCorC       LDVIIESAHSR---------FPVISED-KDHIEGILMAKDLLPFMRSDAEAF---SMDKV   138
TpCorC       LALYREHRYSR---------VPVYRES-VDHIVGVAYAKDLLDYYCEEDLKGRT-V-ASI   294
AtMGR8       HSMWVTHQYSR---------VPVFEQR-IDNIVGIAYAMDLLDYVQKGDLLESTSVGDMA   427
AtMGR9       HNFWVTHQYSR---------VPVFEQR-IDNIVGIAYAMDLLDYVPKGKLLESTTVVDMA   422
hCNNM2       MSEIMESGYTR---------IPVFEGE-RSNIVDLLFVKDLA-FVDPDDCTPLKTITKFY   518
hCNNM4       MSEIMESGYTR---------IPVFEDE-QSNIVDILYVKDLA-FVDPDDCTPLKTITRFY   445

AtMGT10      ----LEVLPNRLTADILEELRISKQRLVELG----SRAGALRQMLLDL-------LEDPH   310
EcCorA       RDGRLFTLRERELP-AFRLYRMRARSQSM-------VDGNAYELLLDLFETKIEQLADEI   148
EcCorC       LRQAVVVPESKRVDRMLKEFRSQRYHMAIVI--------DEFGGVSGL----- T EDIL   185
TpCorC       THPPYFVPENMDAWSLLKELRRRKVHMAIVV--------DEFGGTAGL----- T EDVI   341
AtMGR8       HKPAYFVPDSMSVWNLLREFRIRKVHMAVVL--------NEYGGTIGI----- T EDVV   474
AtMGR9       HKPAFFVPDSMSVWNLLREFRIRKVHMAVVL--------NEYGGTIGI----- T EDVV   469
hCNNM2       NHPLHFVFNDTKLDAMLEEFKKGKSHLAIVQRVNNEGEGDPFYEVLGI----- T EDVI   573
hCNNM4       NHPVHFVFHDTKLDAMLEEFKKGKSHLAIVQKVNNEGEGDPFYEVLGL----- T EDVI   500
.  .      :.  :                          .:         : *

AtMGT10      E--------IRRICIMGRNCTLRRGDDDLECTLPSDKLIAEEEEEEIEMLLENYLQRCES   362
EcCorA       ENIYSDLEQLSRVIMEGHQG---DEYDEALSTL------AELEDI---------------   184
EcCorC       E-----------LIV-GEIE---DEYDEEDDID-----FRQLSRH---------------   210
TpCorC       E-----------EIV-GEIY---DETDEPEDAA-----IRRLPDG---------------   366
AtMGR8       E-----------EIV-GEIF---DENDSKEEIQKKTGYIVMRDEG---------------   504
AtMGR9       E-----------EIV-GEIF---DENDSKEEIQKKTGYIVMRAEG---------------   499
hCNNM2       E-----------EIIKSEIL---DETDLYTDNRTKKKVAHRERKQ---------------   604
hCNNM4       E-----------EIIKSEIL---DESDMYTDNRSRKRVSEK-NKR---------------   530
*                 : ..        *

AtMGT10      CHGQAERLLDSAKEMEDSIA-----VNLSSRRLEVSRFELLLQ-----------------   400
EcCorA       --GWKVRLC--LMDTQRALNFLVRKARLPGGQLE-QAREILRDIESLLPHN---------   230
EcCorC       --TWTVRALASIEDFNEAFG-----THFSDE-----------------------------   234
TpCorC       --SLSIQAQTPIDEVSEALG-----VELPEG-----------------------------   390
AtMGR8       --IYDVDANTSIDQLSEELN-----MKMPEGI----------------------------   529
AtMGR9       --IYDVDANTSIDQLSEELN-----IKMAEGH----------------------------   524
hCNNM2       -------DFSAFKQTDSEMK-----VKISPQLLLAMHRFLATEVEAFSPSQMSEKILLRL   652
hCNNM4       -------DFSAFKDADNELK-----VKISPQLLLAAHRFLATEVSQFSPSLISEKILLRL   578
: .  :       .:

AtMGT10      ---------------VGTFCV-------------------------AVGALIAGIFGMNL   420
EcCorA       --------ESLFQKVNFLMQAAMGFINIEQNRIIKIFSVVS--VVFLPPTLVASSYGMNF   280
EcCorC       ----------EVDTIGGLVMQAFGHLPARG-ETIDI--DGYQFKVAMADS----------   271
TpCorC       ----------EYDTLSGFLYALFGRIPSVG-ESVEW--QGFRFVVE--------------   423
AtMGR8       ----------QYETVSGFVCEAFGYIPKTG-ESVKV--VLEKESWEEDGEEEEGKQERQE   576
AtMGR9       ----------QYETVSGFVCEAFGYIPKTG-ESVTV--VLEKENWEENDEQDEGKHERQD   571
hCNNM2       LKHPNVIQELKYDEKNKKAPEYYLY--QRN-KPVDYFVLILQGKVE----VEAGKEGMKF   705
hCNNM4       LKYPDVIQELKFDEHNKYYARHYLY--TRN-KPADYFILILQGKVE----VEAGKENMKF   631

AtMGT10      RSYLEEQASAFWLTTG---GIIIGAAVAFFLMYSYLS-RRKIF-----------------   459
EcCorA       EFMPEL-KWSFGYPGAIIFMILAGLAPYLYFK------R---------------------   312
EcCorC       --------------------------RRIIQV------HVKIPDDSPQP-----------   288
TpCorC       ----------------------SADQRRIERV------RVERLVEHGEG-----------   444
AtMGR8       -----P-KEKNQIYRV---EILAGNARKVSAV------RFERVNDMDQVSEASDVKSMVP   621
AtMGR9       -----Q-KEKHQIYRL---EILAGNARKVSAV------RFERVSDMDQVSEARDVKNMVP   616
hCNNM2       -----E-ASAFSYYGV---MALTASPVPLSLSRTFVVSRTELLA-AGSPGENKSPPRPCG   755
hCNNM4       -----E-TGAFSYYGT---MALTSVPSD------------------------RSPAHPTP   658

AtMGT10      ------------------------------------------------------------   459
EcCorA       ----KNWL----------------------------------------------------   316
EcCorC       K-LDE-------------------------------------------------------   292
TpCorC       ------------------------------------------------------------   444
AtMGR8       K-FVRKWSSEEDDGNLSNEEDQSENAVLDEHVLA----DNS--------------KKQQ-   661
AtMGR9       K-FVRKWSSEEDSDG----NLQAKNAVFDEHLIA----ETE--------------SMKKE   653
hCNNM2       LNHSDSLSRSDRIDAV-TPTLGSSNNQLNSSLLQVYIPDYSVRALSDLQFVKISRQQYQN   814
hCNNM4       LSRSASLSYPDRTDVSTAATLAGSSNQFGSSVLGQYISDFSVRALVDLQYIKITRQQYQN   718

AtMGT10      ------------------------------------------------------------   459
EcCorA       ------------------------------------------------------------   316
EcCorC       ------------------------------------------------------------   292
TpCorC       ------------------------------------------------------------   444
AtMGR8       ------------------------------------------------------------   661
AtMGR9       ------------------------------------------------------------   653
hCNNM2       ALMASRMDKTPQSSDSENTK-IELTLTELHDGLPDETANLLNEQNCVTHSKANHSLHNEG   873
hCNNM4       GLLASRMENSPQFPIDGCTTHMENLAEKSELPVVDETTTLLNERNSLLHKASHEN-----   773
```

```
AtMGT10      --          459
EcCorA       --          316
EcCorC       --          292
TpCorC       --          444
AtMGR8       --          661
AtMGR9       --          653
hCNNM2       AI          875
hCNNM4       AI          775
```

Supplementary Figure S1. Phylogenetic tree and protein sequence alignment of *Arabidopsis thaliana* MGT10, MGR8 and MGR9, *Escherichia coli* CorA and CorC, *Thermus parvatiensis* CorC and *Homo sapiens* CNNM2 and CNNM4. (**A**) The tree was generated using the neighbor-joining method with 500 bootstraps and the default settings of MEGA11. Numbers above branches indicate posterior probability values and the expected number of changes per site along the branches is indicated by the scale bar. (**B**) Protein sequence alignment was performed using Clustal Omega https://www.ebi.ac.uk/Tools/msa/clustalo/. The sequences of the DUF21, CBS-pair, and CorC-HlyC domains are colored in blue, red, and yellow, respectively. Residues within these domains which were mutated and analyzed heterologously in *E. coli* are highlighted in the respective background. The TpCorC Asn^{94} residue is highlighted in black, the chloroplast-targeting peptides of MGR8/9 are shown in green, the Gly-Met-Asn (GMN) motif is highlighted in yellow, the peptide sequence used as epitope for raising the anti-MGR8 antibody is highlighted in pink.

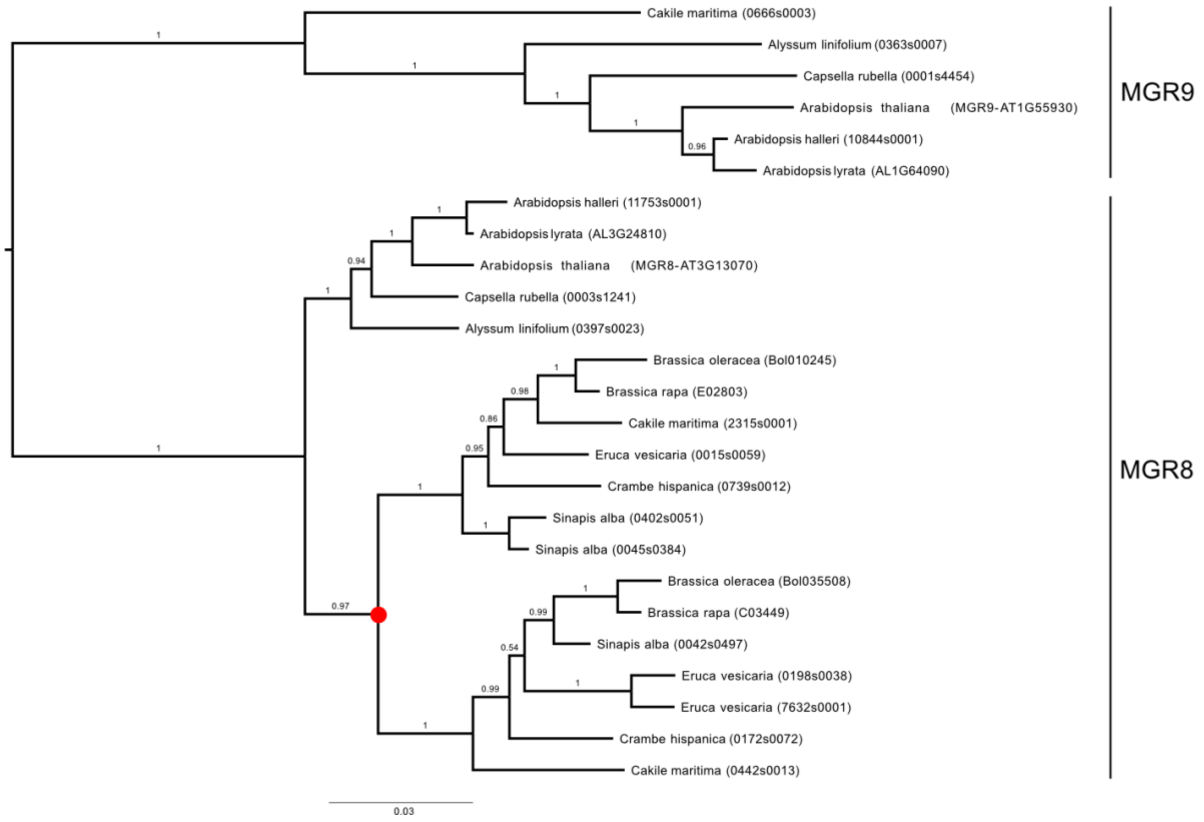

Supplementary Figure S2. Diversification of MGR8 and MGR9 transporters in the plant family *Brassicaceae*. The phylogenetic tree was constructed using MrBayes v3.2.6 The MGR8 and MGR9 types of transporters form two well supported clades, and species from *Arabidopsis*, *Capsella,* and *Alyssium* harbor both types. In *Brassica*, *Eruca*, *Crambe,* and *Sinapis,* only the MGR8 type was found, albeit in two or more versions separated into two well-supported sub-clades, resulting from a gene duplication (indicated by a red dot). *Cakile maritima* stands out in this analysis by coding for MGR9 and two sub-variants of MGR8. Numbers above branches indicate posterior probability values and the expected number of changes per site along the branches is indicated by the scale bar.

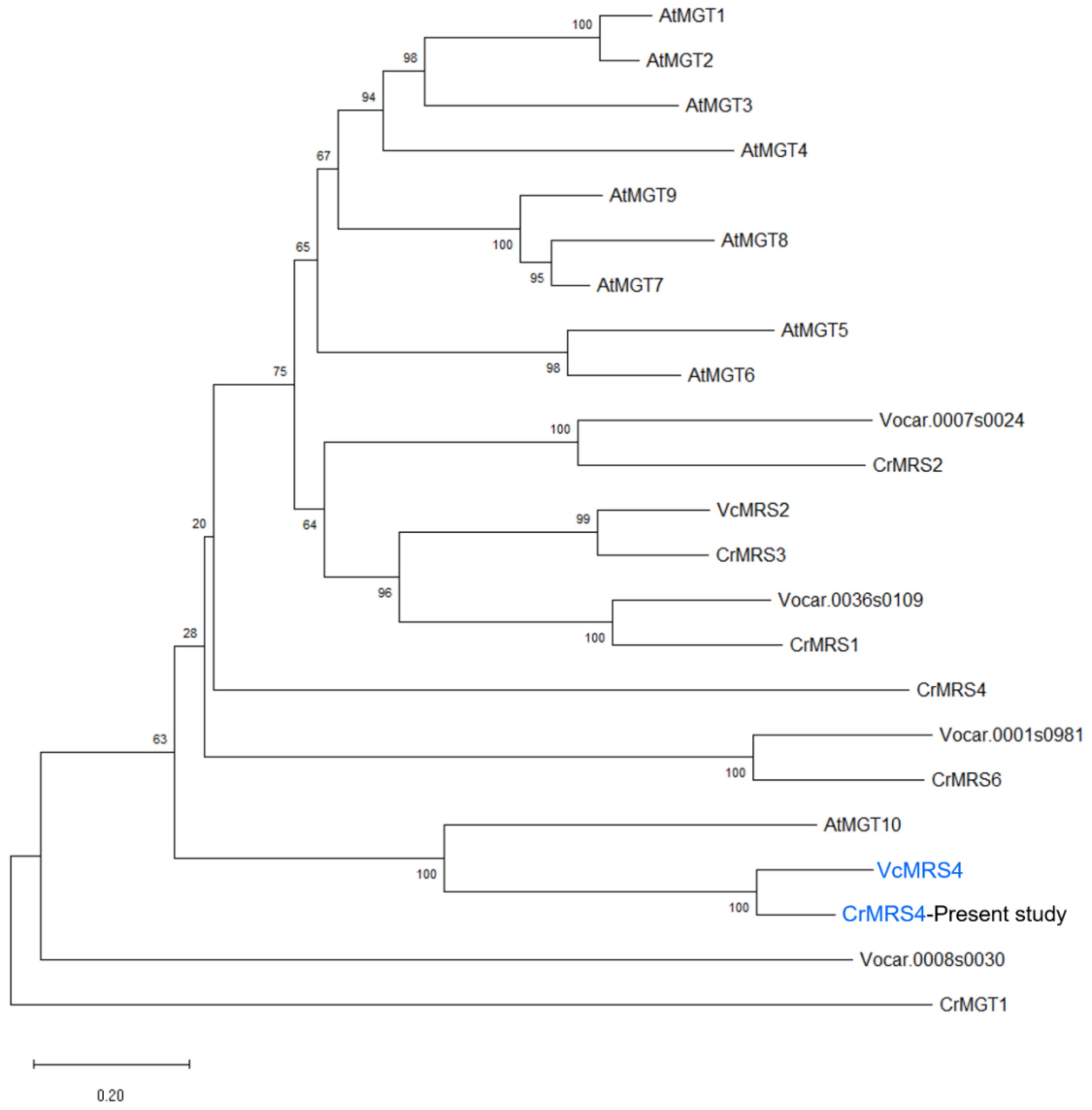

Supplementary Figure S3. Phylogenetic tree of protein sequences from *Arabidopsis thaliana*, *Chlamydomonas reinhardtii*, and *Volvox carteri* that are homologs of the Arabidopsis MGT10. The tree was generated using the neighbor-joining method with 500 bootstraps and the default settings of MEGA11. Closest homologs of MGT10 are highlighted in blue. Numbers above branches indicate posterior probability values and the expected number of changes per site along the branches is indicated by the scale bar.

```
AtMGT10    --------------------MALTPIPSTFTSLFNFSDHSPYPSPSL-------------   27
CrMRS4     ------------------------------------------------------------   0
VcMRS4     MNKGLLVPKEVNVTCRRHDVAQHLSGPAARRRCFARSRYGFYPWEHIDKCSHASRLATLP   60

AtMGT10    ------------HYLLPGS------SPSFSLQLSALSRTPIYFEALKVLSRSKCFAKSPT   69
CrMRS4     ----------------------------------MVQSANQILHQLEAANAATQAA-NGG   25
VcMRS4     TRPAAGSRGFDEARWLPGQRFVPQCSSSHNTAEEMMQSANQILHQLEAAGAATHAAQHAG   120
                           :. :   :. *:. . :.  *

AtMGT10    TAEDFVGDYESLNVS----DDDDGSDS-NSSDG-DNGGGRDDSKKIDSS-----------   112
CrMRS4     VNNTIVSGYSSSSGSMSPLQN-----------------SADSMSSMDEYST-SMDFSDLA   67
VcMRS4     NNGSIVSGYASSTGSMSSTYNNNSSSSKDSRDSRDSRDGGDSLSSMDEYSSSTLDFSDLA   180
                :*..* * . *     :                 . *. ..:*.

AtMGT10    -----SSSSSSDSTSLGIREPVYEVVEVKATGAISTRKINRRQLLKSSGLRPRDIRSVDP   167
CrMRS4     NEEAL-DAGKGDGAAGGYGKANYEVLRLDATARARRFFVRRRDLLREHRLQPRDLRRIDP   126
VcMRS4     NEDAVADAAKGEGIAGAYGKANYEVLRLDATARARRFFVRRRDLLREHRLQPRDLRRIDP   240
           .:...:. : .  :  ***:.:.**.      :.**:**:.  *:***:* :**

AtMGT10    SLFMTNSVPSLLVREHAILLNLGSLRAIAMRDRVLIFDYNRRGGRAFVDTLMPRLNPRSM   227
CrMRS4     SIDFTKTSPSITIKEDVLLLNLGGVRAIVTAEKALLFEPNSATTRKFLEVVAPRLQTHGA   186
VcMRS4     SIDFTKTSPSITIKEDVLLLNLGGVRAIVTAEKALLFEPNSATTRKFLEVVLPRLQTHGQ   300
        *: :*:: **: ::*..:*****.:***.  ::.*:*: *    * *::.: ***: :.

AtMGT10    N---------------------------------GGPSMPFELEAVESALISRIQRLEQR   254
CrMRS4     ARQQAASRGDPAAYVNVSHADYMARFYHQGKFDSDSRTPPFELEVLEGALTVAVGRLDAE   246
VcMRS4     ARQQALMRGPPSAYVNVSHADYMARFYYQGKFDSSSRTPPFELEVLEGALMVAVGRLDAE   360
                         .. : *****.:*.**   : **: .

AtMGT10    LMDIEPRVQALLEVLPNRLTADILEELRISKQRLVELGSRAGALRQMLLDLLEDPHEIRR   314
CrMRS4     MAGVTERVSALLTKLPGDITPVNLEELRRVKQALVELENKADTLREMLEELMDDEDELRE   306
VcMRS4     MSGVTDRVSALLTKLPGDITPVNLEELRRVKQALVELEDKADTLREMLEELMDDEDELRE   420
        : .:  **.***  **. :*   *****  ** **** .:*.:**:** :*::* .*:*.

AtMGT10    ICIMGRNCTL--------------------------------------------------   324
CrMRS4     LNLSSRPRREDRRRQRERNRLEREVERAREIKEELEERALDDGQPITAALPPAPLPPPPP   366
VcMRS4     LNLSSRPRREDRRRQRERNRLEREVERAREIKEELEERALDDGQPITAALPMPPMPMSMP   480
                                   : : .*

AtMGT10    ------------------------------------------------------------   324
CrMRS4     GGGAAAAAAAAAGIAGLPGQGLGGPGLGPGAGMGPAAMPV-----SAAGMGPSILGPGAP   421
VcMRS4     PATPPLTSTAAAAAASPPGTGADGGSVTLYGPYGPVGPPPSALSPGAAKLNGYVSPPGLN   540

AtMGT10    ------------------------------------------------------------   324
CrMRS4     GAGGGGGGAATAGVSPYSPYGPNAVPTVAPPAMSPGLPVAPPPQRVNGHYVYTPPQPQQH   481
VcMRS4     GSGSGGGGYY-------------------------------------------PHIP---   554

AtMGT10    ------------------------------------------------------------   324
CrMRS4     TPPVTHIGGSVAAGLGIGMAPGARPGAAAAPPPPPAPPPGSV---------YP--MLGPY   530
VcMRS4     --GVS--GVAVVRGPGGV----YSPG-GLAPPPPPAPLPSSAVGGGNGVVAYPPMQGGPY   605

AtMGT10    --------------------------------------------RRGDDD----------   330
CrMRS4     SGNTV-------GSNPARQERLRELRTKYDRDRLRELRARFGWVREGREDGTGGSLGGGA   583
VcMRS4     LGESAAAAAGRREPTTARQERLRELRTKYDRERLRELRARFGWVREGRDDSGAAA-----   660
                                   *.* :*

AtMGT10    ------------------------------------------------------------   330
CrMRS4     AAAGGGGGGGYAAGGGFAAAGGSVALGGAGRTAGGGYSLGRGRSSSASGGKSRGDKSRGE   643
VcMRS4     ---GSLGSSGVAA------------------------------------AAAAGTATAAG   681

AtMGT10    ------------------------------LECT-LPSDKLI----AEEEEEEIEMLLEN   355
CrMRS4     GRGEERERDRRKELIEESDYMSRLDDSEEDLQEAQDALEEMVEEEEEEAELEEVEDLLEF   703
VcMRS4     VRKEERERDRRKELIEEADFMSRLGDSEEDLQEAQDALEEMVEEEEEEAELEEVEDLLEF   741
                       *: :    ::::     * * **:* ***

AtMGT10    YLQRCESCHGQAERLLDSAKEMEDSIAVNLSSRRLEVSRFELLLQVGTFCVAVGALIAGI   415
CrMRS4     YLQRASALQSEAERMLAGARDLEESIGVSLSARRYEVNRLELMLSIGSFAAAIGAMLAGI   763
VcMRS4     YLQRASGLQSEAERMLAGARDLEESIGVSLSARRYEVNRLELMLSIGSFAAAVGAMLAGI   801
        ****...    :.:***:* .*:::*:**.*.**:** **.*:**:*.:*:*..*:**::***

AtMGT10    FGMNLRSYLEEQASAFWLTTGGIIIGAAVAFFLMYSYLSRRKIF   459
CrMRS4     FGMNMRSNLEHSMLSFWGISGAIVLGCAWIFFAVMRYTRSKRIL   807
VcMRS4     FGMNMRSNLEQSAVSFWGVTAAIVLGCAWIFFAVMRYTRSKRIL   845
         ****:** **..  :**  :..*::*.*  ** :  *   ::*:
```

Supplementary Figure S4. Protein sequence alignment of AtMGT10, CrMRS4, and VcMRS4. Performed using Clustal Omega https://www.ebi.ac.uk/Tools/msa/clustalo/. The transmembrane domains are determined using TMHMM and are colored in blue. The chloroplast-targeting peptide of MGT10 is shown in green. The Gly-Met-Asn (GMN) motif is highlighted in yellow.

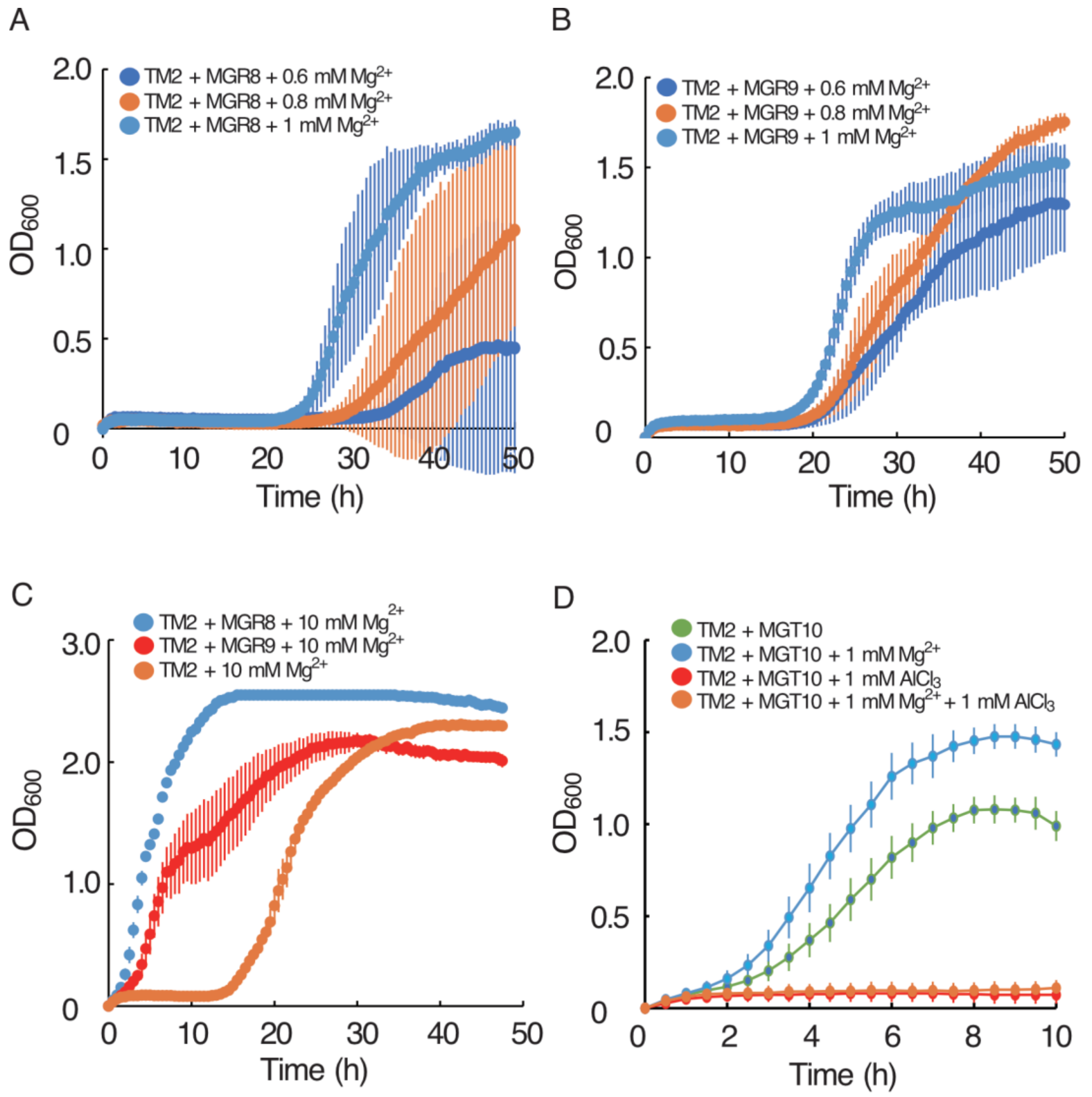

Supplementary Figure S5. Growth of the *E. coli* TM2 transformed with the plasmid containing *MGR8* (**A**, **C**), *MGR9* (**B**, **C**), and *MGT10* (**D**) cDNA. (**A, B**) Effects of Mg^{2+} concentration in LB medium. Cells were grown at 37 °C on LB medium supplemented with different concentrations of $MgSO_4$. LB medium without added $MgSO_4$ contained 0.17 mM Mg^{2+} (Ishijima et al., 2015). (**C**) Cells were transformed with the plasmid containing *MGR8* and *MGR9* cDNA and with the pTV118N vector. Cells were grown at 37 °C on LB medium supplemented with 10 mM $MgSO_4$. (**D**) Al^{3+} inhibition of growth of the *E. coli* TM2 transformed with the plasmid containing *MGT10* cDNA. Cells were grown at 37 °C on LB medium supplemented without and with 1 mM $MgSO_4$. $AlCl_3$ was added at 0 mM and 1 mM concentration. The OD_{600} was measured every 0.5 h. Data are average values of three or more independent experiments, and error bars indicate means ± S.E.M. Where not visible, error bars are smaller than the symbols.

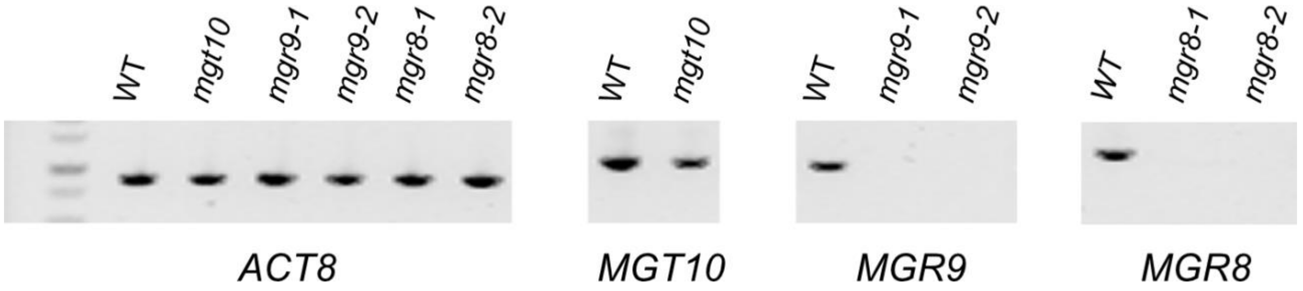

Supplementary Figure S6. RT-PCR analysis of *mgt10* and *mgr* mutants. Wild type (WT) was used as a positive control and *ACT8* as a reference gene. Detection of the *MGT10* transcript at a lower intensity than in WT together with the observed characteristic yellow vein phenotype (see Supplementary Fig. S9A) indicate that the *mgt10* line is a knockdown mutant. Absence of the corresponding transcript in the *mgr* lines indicates that they are knockout mutants.

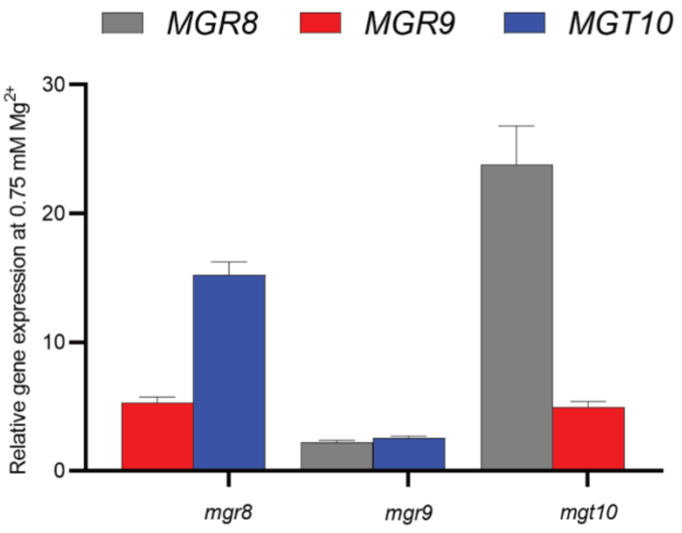

Supplementary Figure S7. Expression pattern of *MGR8*, *MGR9*, and *MGT10* in Arabidopsis mutants. Two lines of *mgr8*, two lines of *mgr9*, and the *mgt10* mutant were grown hydroponically with 0.75 mM Mg^{2+}. Relative expression levels in leaves of 6-week-old plants were determined using quantitative RT-PCR. *ACTIN8* and *PEX4* were used as internal standards.

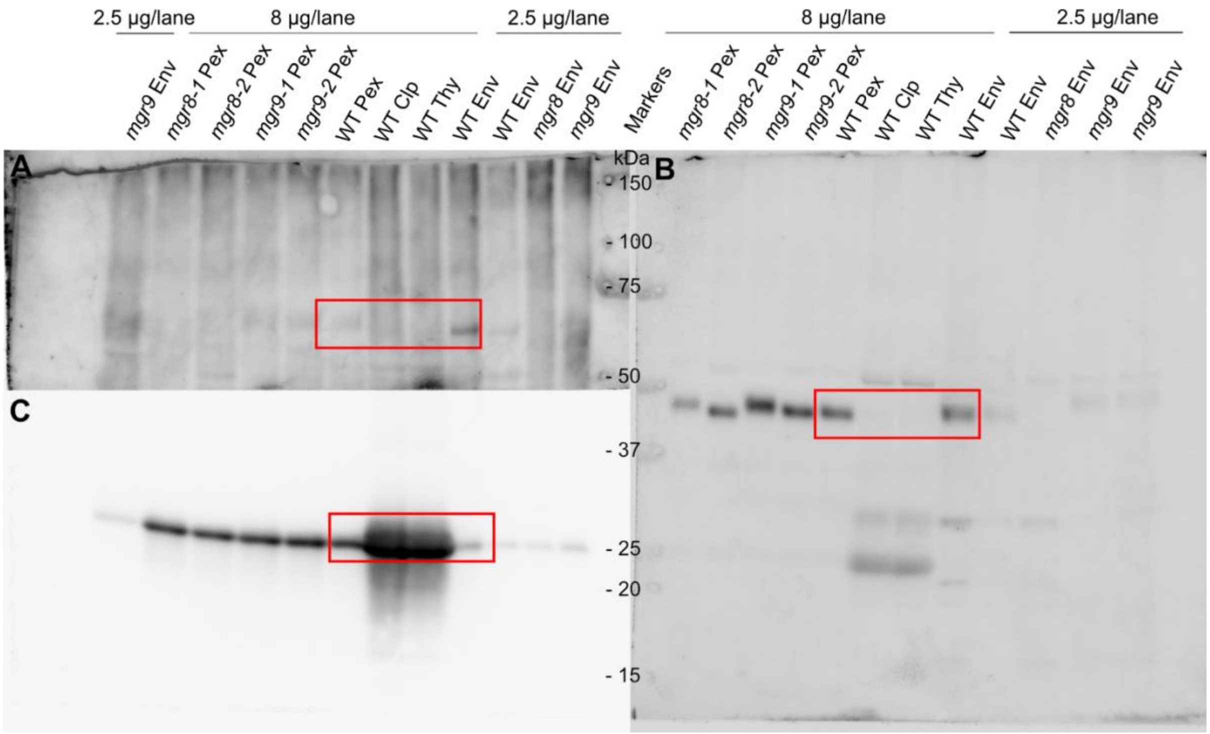

Supplementary Figure S8. Uncropped version of the immunoblots shown in Figure 3D. Total protein extracts were prepared from Arabidopsis wild-type (WT) plants, and two lines each of *mgr8* and *mgr9* mutants. Chloroplasts (Clp), thylakoid (Thy), and envelope (Env) membranes were purified from wild-type leaves and in the case of the envelope also from *mgr8* and *mgr9* mutants. Proteins were separated by SDS-PAGE, transferred to PVDF membranes, and probed with an antibody against MGR8 (**A**). The inner envelope translocon complex Tic40 protein (**B**) and the light-harvesting Chl *a/b* binding thylakoid protein Lhcb1 (**C**) were used for even loading of the protein extract lanes and as marker proteins for the respective compartment. The absence of a cross-reacting band in the *mgr8* mutant lanes confirms the specificity of the raised antibody.

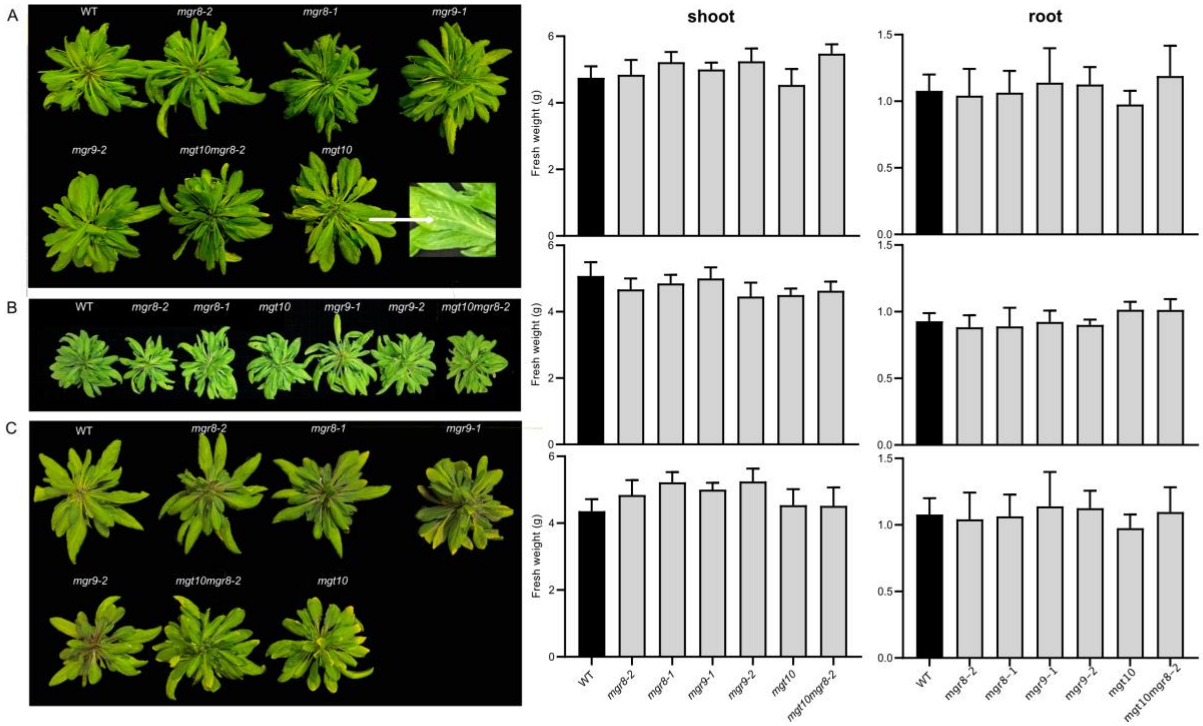

Supplementary Figure S9. Growth and biomass of the Arabidopsis mutants used in this work. Wild-type (WT) plants and mutants were grown hydroponically at 0 mM (**A**), at standard (0.75 mM) (**B**), and at 3 mM (**C**) Mg^{2+}. Representative photos and fresh weight of shoots and roots of the 6-7 week-old plants are shown. The plotted data are means ± S.E.M. (n = 8-10 plants). There were no significant differences among the genotypes at any of the tested conditions according to Tukey one-way ANOVA ($P > 0.05$).

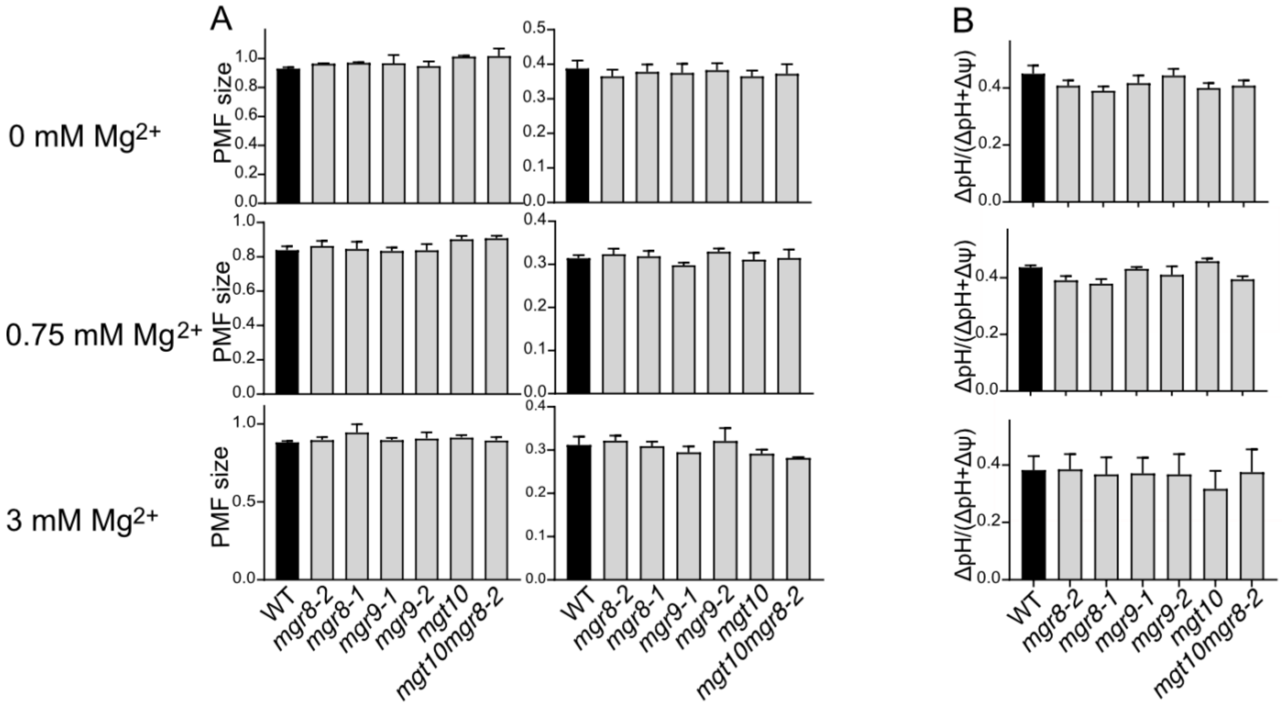

Supplementary Figure S10. Proton motive force (PMF) size and partitioning to ΔpH for the experiments presented in Figure 5, A–C. (**A**) PMF size left- and right panels are for low-to-high light and high-to-low light transitions, respectively. (**B**) PMF partitioning to ΔpH is for high-to-low light transitions. The plotted data are means ± S.E.M. (n = 4-7 plants). There were no statistically significant differences among the genotypes according to Tukey one-way ANOVA ($P > 0.05$).

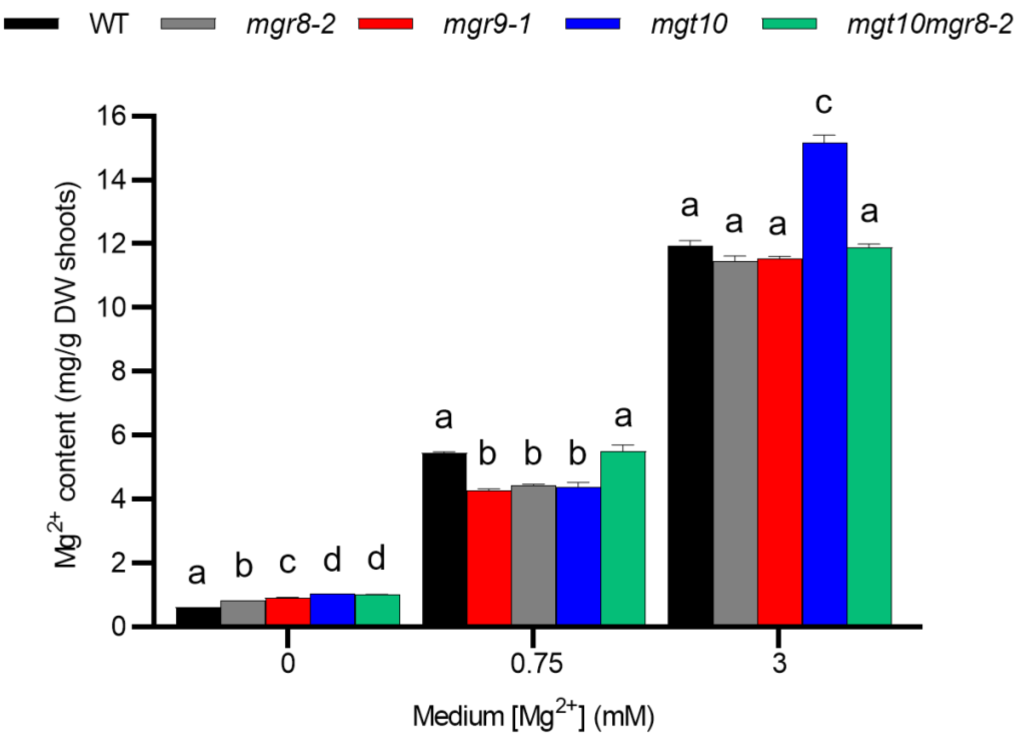

Supplementary Figure S11. Mineral content in Arabidopsis shoots. Wild type (WT) plants and mutants were grown hydroponically at the indicated $MgSO_4$ concentrations. Mg^{2+} content was determined in shoots using ICP-OES. The data presented are expressed as means ± S.E.M. (n = 3 shoots). Different letters indicate statistically significant differences among the genotypes according to Tukey one-way ANOVA ($P < 0.05$).

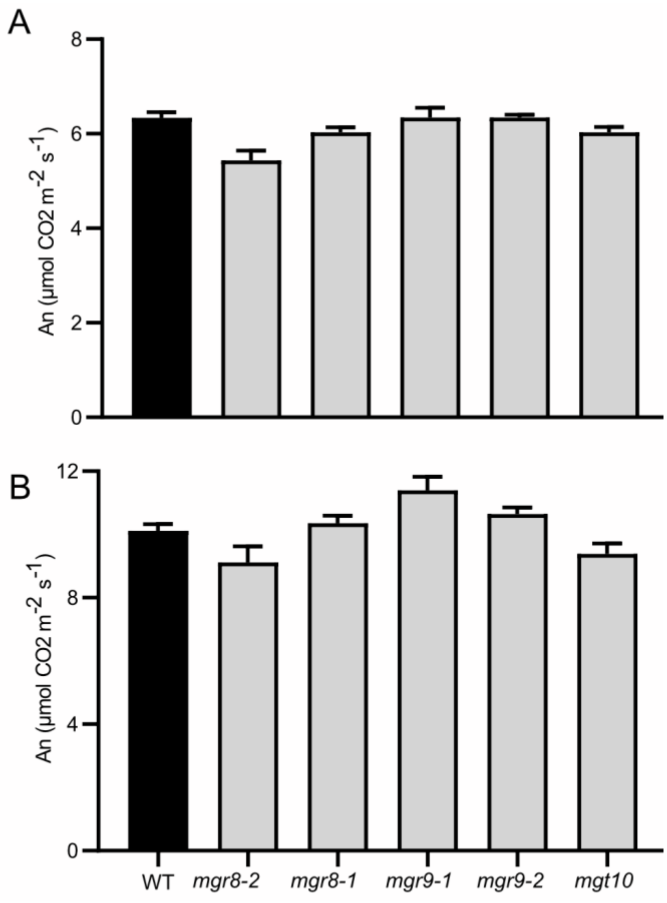

Supplementary Figure S12. Net photosynthesis in Arabidopsis. Wild-type (WT) plants and mutants were grown at 120 µmol photons m^{-2} s^{-1} and carbon fixation in terms of net photosynthesis (A_n) was measured at atmospheric CO_2 concentration during illumination for 5 min at 120 µmol photons m^{-2} s^{-1} (**A**) and 660 µmol photons m^{-2} s^{-1} (**B**). The data are expressed as means ± S.E.M. (n = 6). There were no significant differences among the genotypes at any of the tested conditions according to Tukey one-way ANOVA ($P > 0.05$).

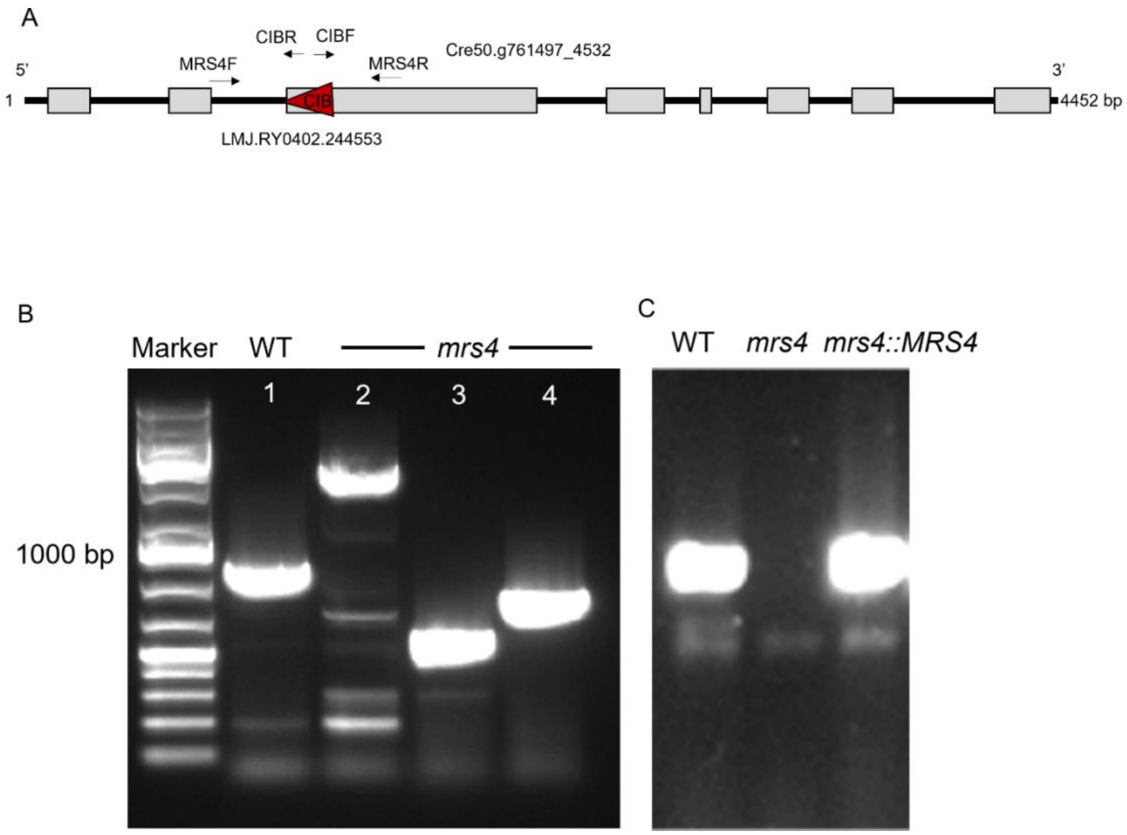

Supplementary Figure S13. CIB mutant confirmation. (**A**) The position of the CIB insert in the *MRS4* gene and the PCR reactions used to confirm the insert in the CLiP *mrs4* mutant. (**B**) The insert position was confirmed with primers specific for the insert (CIBF/R) and to the gene (MRS4F/R). Lane 1 and 2, MRS4F and MRS4R primers using DNA from WT and *mrs4*, respectively; lane 3, CIBF (insert) and MRS4R primers using *mrs4* DNA; lane 4, CIBR (insert) and MRS4F primers using *mrs4* DNA as template. The size difference between lanes 1 and 2 indicates the presence of the CIB cassette of about 1800 bp. (**C**) The *mrs4* was transformed with the Volvox *MRS4*. The *mrs4::MRS4* is a complemented line as confirmed with specific primers.

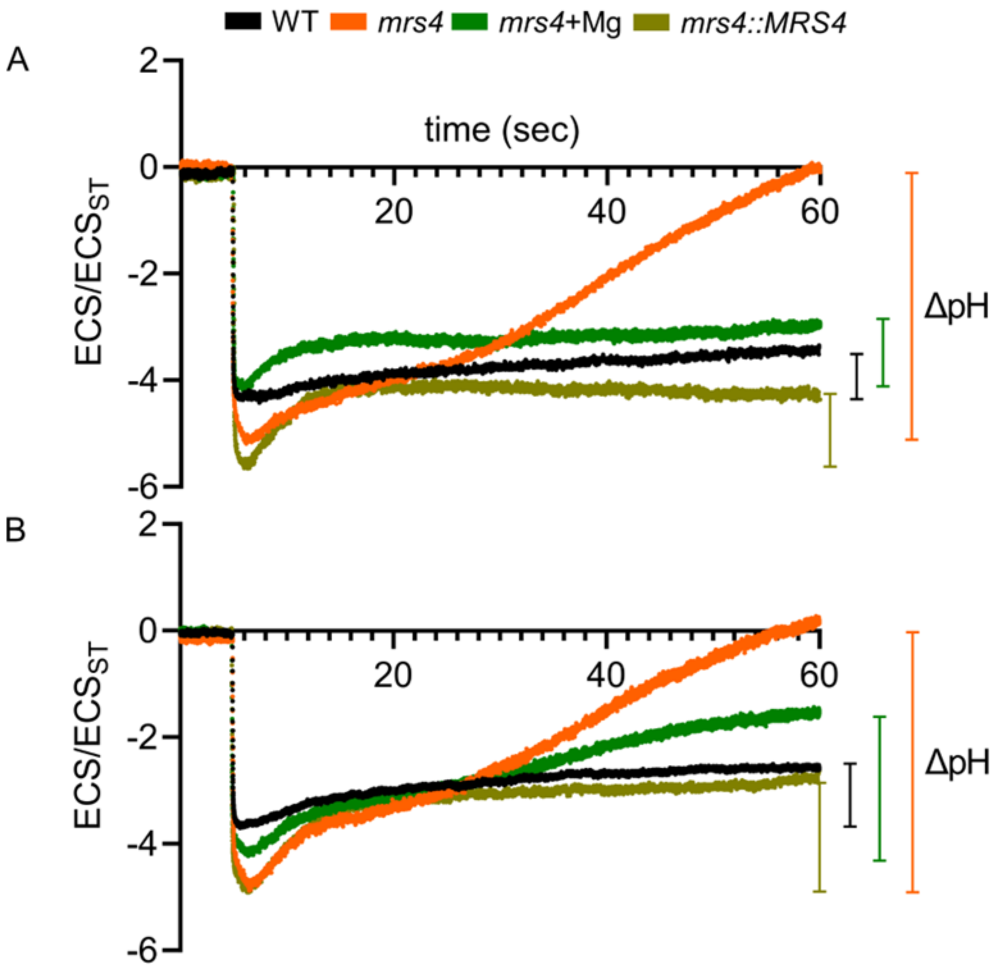

Supplementary Figure S14. Electrochromic shift (ECS) decay analysis. Wild type (WT), *mrs4*, and complemented *mrs4:MRS4* were grown on TAP liquid medium in darkness and where indicated supplemented with 5 mM Mg^{2+}. The cells were dark-adapted for 20 min and then illuminated for 2 min (**A**) or 7 min (**B**) at 660 µmol photons m^{-2} s^{-1} after which the light was switched off to record ECS in darkness. The coloured bars show the PMF partitioning to ΔpH.

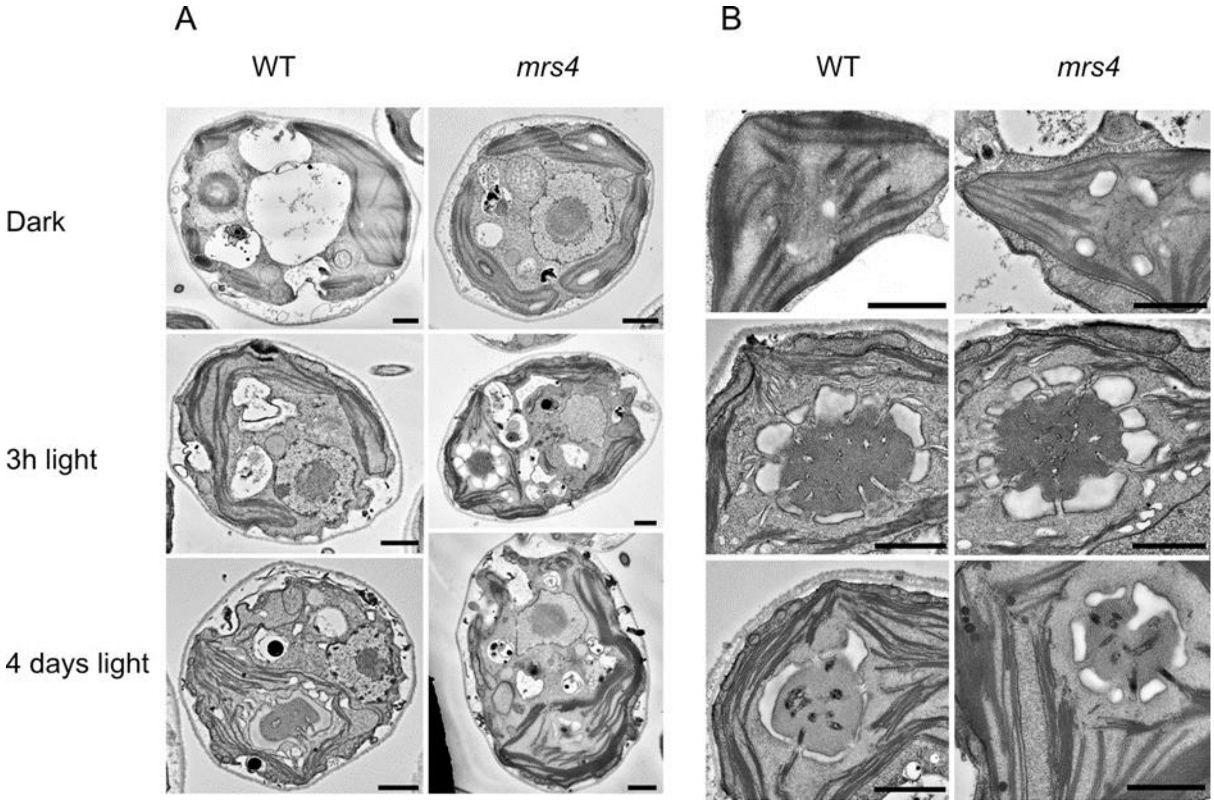

Supplementary Figure S15. Ultrastructure of the Chlamydomonas cell (**A**) and chloroplast (**B**). Wild type (WT) and *mrs4* mutant were grown on liquid TAP medium in darkness for 4 days or on TP medium in continuous light (100 µmol photons m^{-2} s^{-1}) for 3 h or 4 days and samples were fixed for electron microscopy as described in Methods. Scale bar: 1 µm. While the mutant chloroplast displays a WT-like ultrastructure in dark and 3 h light, after 4 days in the light, the thylakoid stacks are longer and consist of more layers than in WT. In all three conditions, the *mrs4* chloroplasts contain slightly larger and more numerous starch grains than in WT.

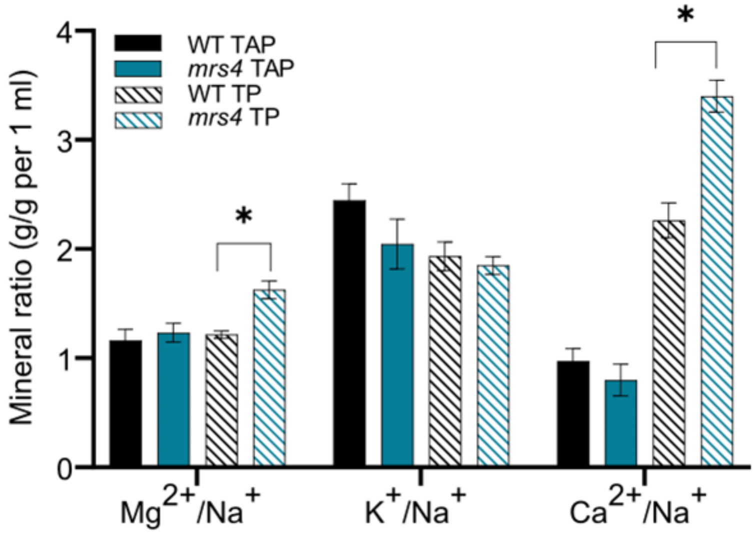

Supplementary Figure S16. Mineral ratio of Chlamydomonas cells. Wild type (WT) and *mrs4* mutant were grown for 4 days on either liquid TAP medium in darkness or on TP medium in continuous light (100 µmol photons m^{-2} s^{-1}). The cells were washed, and the mineral content was determined by ICP-MS. The plotted data are means ± S.E.M. (n = 3 replicates). Asterisks indicate statistically significant differences between WT and mutant according to Student's t-test ($P < 0.05$). The *mrs4* mutant displays enhanced Mg^{2+} and Ca^{2+} content when grown in light conditions.

Supplementary Tables

Supplementary Table S1. F_v/F_m of the mutants used in this work.

**F_v/F_m**	**WT**	***mgr8-2***	***mgr8-1***	***mgr9-1***	***mgr9-2***	***mgt10***	***mgt10mgr8-2***
**0 mM**	0.836±0.001	0.875±0.001	0.828±0.002	0.815±0.001*	0.8134±0.003*	0.803±0.002*	0.832±0.003
**0.75 mM**	0.823±0.005	0.818±0.005	0.828±0.004	0.815±0.002	0.787±0.033	0.801±0.007	0.835±0.003
**3 mM**	0.796±0.001	0.797±0.003	0.801±0.002	0.781±0.031*	0.808±0.002	0.741±0.021	0.816±0.033

Wild-type (WT) plants and mutant lines were grown for 6-7 weeks at 0 mM, 0.75 mM, and 3 mM Mg^{2+}. Fast Chl fluorescence kinetics were recorded on 30 min dark-adapted plants and the F_v/F_m was calculated as described in Methods. The plotted data are means ± S.E.M. (*n* = 10 plants). Asterisks indicate statistically significant differences between WT and each of the mutants according to Tukey one-way ANOVA ($P < 0.05$).

Supplementary Table S2. Primers used in this work.

**Gene name (Accession number)**	**Application**	**Forward sequence**	**Reverse sequence**
AtMGR8 (At3g13070)	PCR, *mgr8-1*	AGTTGCCGCAATATTCACAAC	ACTGAGTAAGGAGGAGCCGTC
	PCR, *mgr8-2*	AAAATCATGGAGGCCATAAGC	AATTGAGTGGTGCCATTGAAG
	RT-PCR, *mgr8-1, mgr8-2*	GGGTATGGCACTTGAATTATCTGTT	GTCTCGTACTGAATGCCCTCTGGC
	TM2 complementation	AGGAAACAGACCATGTTTATGCTGTTTAGCTCCAGTC	ATGCCTGCAGGTCGACTCATTGTTGTTTCTTGCTATTATCA
	Mutagenesis, E261K	AGATTACTCCAAAAAGTGTTGCTGTGC	TAGTCAGGAGTAAAATAGCCACTGTC
	Mutagenesis, R388L	CTGGTGCCTGTTTTTGAGCAGCGTATTG	TGAGTACTGATGGGTCACCCACATACTATGG
	Mutagenesis, P390A	TTGAGCAGCGTATTGATAATATAGTGGGAATTGCATATGC	AAACAGCCACCCTTGAGTACTGATGGG
	Mutagenesis, P390L	TTGAGCAGCGTATTGATAATATAGTGGGAATTGCATATGC	AAACAAGCACCCTTGAGTACTGATGGG
	Mutagenesis, G466A	CAATAGTAACTCTTGAAGATGTGGTCG	CGATCGTTCCACCATATTCGTTAAG
	Mutagenesis, T469A	GCTCTTGAAGATGTGGTCGAAGAGATTGTTGG	TACTATTCCGATCGTTCCACCATATTCGTTAAGGAC
	Mutagenesis, T469I	TTCTTGAAGATGTGGTCGAAGAGATTGTTGG	TACTATTCCGATCGTTCCACCATATTCGTTAAGGAC
	Mutagenesis, E471A	CAGATGTGGTCGAAGAGATTGTTGG	CAAGAGTTACTATTCCGATCGTTCCAC
	Mutagenesis, D472A	CTGTGGTCGAAGAGATTGTTGGTG	CTTCAAGAGTTACTATTCCGATCGTTCC
AtMGR9 (At1g55930)	PCR, *mgr9-1*	CTTTAGTGACCAAGGCAGTCG	ATTAAACCGGTCTGGTATGGC
	PCR, *mgr9-2*	TGGCATTACTGAATCCAGGAG	AGAGCCACTTACCAACACTGC
	RT-PCR, *mgr9-1, mgr9-2*	GGCGTTAACTCCAATTCCTTCA	GCCGAATATACCTGCGATGAGA
	TM2 complementation	AGGAAACAGACCATGTTGAGTGCGACTGGAGATC	TTGCATGCCTGCAGGTCGACCACCTCTTTCCTCTTTTATC
AtMGT10 (At5g22830)	PCR, *mgt10*	ATAATAACGCTGGCGGACATCTACATTTT (T-DNA)	TGTTATTTAGGTCCGTGAACATGC
	RT-PCR, *mgt10*	GGCGTTAACTCCAATTCCTTCA	GCCGAATATACCTGCGATGAGA
AtACT8 (At1g49240)	RT-PCR control	CTGCCTTGAAGGTTAAAAGACGA	TGTCAGCCATCTCATTTCCATTTTG
CrMRS4 (Cre50.g761497)	PCR, *mrs4*	ACATGAGGTAAGGCCGTGAG	CCGCTTTCGATATGGTCTGT
VcMRS4 (Vocar.0040s0086)	Cloning	GCTACTCACAACAAGCCCAGTTATGAACAAGGGCCTGCTCGT	CGTGTCCGCCTTGTCCTC
		GAGGACAAGGCGGACACG	GAGCCACCCAGATCTCCGTTAAGGATGCGTTTGCTCCGC
	PCR, *mrs4* complementation	ATGAACAAGGGCCTGCTCGT	GCAACGTTCCACGAACAGC

Supplementary Table S3. Primers for quantitative RT-PCR.

Arabidopsis gene	BioRad unique assay ID	Chromosome location	Amplicon length (bp)
MGR8 (At3g13070)	qAll1CEO0031632	3:4191494-4191598	75
MGR9 (At1g55930)	qAtl1CEO0037997	1:20919288-2091941	93
MGT10 (At5g22830)	qAll1CEO0037470	5:7627930-7628211	111
ACTIN8 (At1g49240)	qAll1CEO0047270	1:18217942-18218083	112
PEX4 (At5g25760)	qAll1CEO0054490	5:8968102-8968288	157
